# Nuclear translocation of ISG15 regulated by PPP2R2B inhibits cisplatin resistance of bladder cancer

**DOI:** 10.1007/s00018-024-05320-1

**Published:** 2024-07-08

**Authors:** Gaowei Huang, Jinwen Liu, Anze Yu, Chenggong Luo, Jiangquan Zhu, Yinghan Wang, Ziran Dai, Lizhen Zhang, Zihao Feng, Jun Lu, Zhong Dong, Junhang Luo, Wei Chen, Zhenhua Chen

**Affiliations:** 1 Department of Urology, The First Affiliated Hospital, Sun Yat-sen University, Guangzhou, 510080 China; 2 Department of Urology, Shenzhen People’s Hospital (the Second Clinical Medical College, Jinan University, the First Affiliated Hospital, Southern University of Science and Technology), Shenzhen, 518020 China; 3 Department of Urology, Guizhou Provincial People’s Hospital, Guizhou University, Guiyang, 550002 China; 4 Department of Urology, Huizhou Central People’s Hospital, Huizhou, 516001 China

**Keywords:** PPP2R2B, Bladder cancer, Cisplatin resistance, ISG15, STING pathway

## Abstract

Cisplatin resistance is a major challenge for systemic therapy against advanced bladder cancer (BC). Little information is available on the regulation of cisplatin resistance and the underlying mechanisms require elucidation. Here, we detected that downregulation of the tumor suppressor, PPP2R2B (a serine/threonine protein phosphatase 2 A regulatory subunit), in BC promoted cell proliferation and migration. What’s more, low PPP2R2B expression was correlated with cisplatin resistance. In vitro and in vivo experiments verified that PPP2R2B could promote BC sensitivity to cisplatin. In terms of mechanism, we identified a novel function of PPP2R2B as a nucleocytoplasmic transport molecule. PPP2R2B promoted ISG15 entry into the nucleus by mediating binding of IPO5 with ISG15. Nuclear translocation of ISG15 inhibited DNA repair, further increasing ISG15 expression through activation of the STING pathway. Besides, PPP2R2B was down-regulated by SUV39H1-mediated histone 3 lysine 9 trimethylation, which could be restored by the SUV39H1-specific inhibitor, chaetocin. Our data suggest that PPP2R2B expression level is a potential biomarker for chemotherapy response and that chemotherapy in combination with chaetocin may be a feasible treatment strategy for patients with BC.

**Supplementary Information:**

The online version contains supplementary material available at 10.1007/s00018-024-05320-1.

## Introduction

Bladder cancer (BC) is a common type of cancer worldwide. Despite the inspiring advances in early detection and diagnosis witnessed in past decades, the prognosis for patients with BC remains bleak [1]. Cisplatin-based chemotherapy is considered the preferred treatment for advanced BC but intratumor heterogeneity and chemoresistance substantially restrict the efficacy of current therapies [2]. Consequently, elucidation of the mechanism underlying cisplatin resistance is important for basic research into BC, as it will facilitate discovery of novel targets related to chemosensitivity and promote progress in precision therapy.

Members of the serine/threonine protein phosphatase 2 A (PP2A) family of tumor suppressors are frequently inactivated in human cancers, and negatively modulate numerous oncogenic pathways in neoplasia [3–5]. PP2A proteins are heterotrimeric complexes comprising a catalytic (C) subunit, a scaffold (A) subunit, and a regulatory (B) subunit, and are involved in metabolism, the cell cycle, and DNA damage responses, among other processes [6]. Enzyme substrate specificity is conferred by the B subunits of PP2A proteins, among which PPP2R2B is a B55 subunit whose inactivation promotes tumor progression and mediates development of tumor drug resistance [4, 7]. Moreover, downregulation of B55 subunits was observed in breast cancer in response to irradiation, while the DNA repair protein, RAD51, was up-regulated [8]. Recently, PPP2R2B has been reported to inhibit bladder cancer progression and serve as a possible biomarker for platinum resistance [9, 10]. However, the molecular mechanism underlying the role of PPP2R2B in bladder cancer and platinum resistance remain largely unknown.

Various biological processes including enhanced DNA repair can contribute to mechanisms underlying platinum resistance [11]. A number of complex molecular classification involved in BC have been reported in recent years, facilitating some progress in the prediction of cisplatin therapy efficacy [12–17]. Here, we show that PPP2R2B can regulate BC proliferation and metastasis. High PPP2R2B expression is negatively related to DNA repair in BC, thus making tumors more sensitive to cisplatin-based chemotherapy. Mechanistically, we identified a novel function of PPP2R2B as a bridging molecule in nucleocytoplasmic transport and determined the upstream molecular regulation of PPP2R2B. Our findings suggest that PPP2R2B has potential to serve as an important biomarker of prognosis and chemosensitivity in BC.

## Materials & methods

### Clinical specimens and cell culture

Surgical specimens from patients with BC were from the Department of Urology, First Affiliated Hospital, Sun Yat-sen University and the informed consent was obtained from each patient. Fresh tumor tissue was rapidly frozen in liquid nitrogen for subsequent extraction of RNA and protein. The J82, 5637, T24, UM-UC-3, and SV-HUC-1 cell lines were purchased from Procell Life Science & Technology (Wuhan, China). HEK-293T cells were purchased from American Type Culture Collection (Manassas, VA). J82 and UM-UC-3 cells were cultured in MEM, 5637 and T24 cells in RPMI-1640, SV-HUC-1 cells in F–12 K, and HEK-293T cells in DMEM media (Gibco, USA), supplemented with 10% fetal bovine serum (FBS) (Excell, China), and incubated in 5% CO_2_ at 37 °C. All cell lines were authenticated by short tandem repeat profiling and routinely tested for mycoplasma infection.

### Antibodies and reagents

Antibodies used for western blotting were as follows: PPP2R2B (ab157461, Abcam), GAPDH (60004-1-Ig, Proteintech), beta tubulin (10068-1-AP, Proteintech), phospho-DNA PKcs (Ser2056) (ab124918, Abcam), phospho-ATR (Ser428) (2853, CST), phospho-ATM (Ser1981) (5883, CST), ISG15 (15981-1-AP, Proteintech), anti-phosphoserine/threonine (PP2551, ECMbio), IPO5 (sc-55,527, Santa Cruz), LaminA/C (10298-1-AP, Proteintech), Human-Reactive STING Pathway Antibody Sampler Kit (38,866, CST), FLAG (F1804, Sigma), HA (51064-2-AP, Proteintech), STAT1 (9172, CST), pSTAT1 (9167, CST), H3 (17168-1-AP, Proteintech), H3K9me3 (ab8898, Abcam), and SUV39H1 (05-615, Sigma). Cycloheximide (GC17198) was from GLPBIO (America); Cisplatin (HY-17,394), LB-100 (HY-18,597) and chaetocin (HY-N2019) were from MedChemExpress (China).

### RT-qPCR

Total RNA was isolated using TRIzol reagent (Thermo, USA) and then reverse-transcribed into cDNA using a 4×EZscript Reverse Transcription Mix II (EZBioscience, USA), following the manufacturer’s instructions. RT-qPCR was performed using 2×SYBR Green qPCR Master Mix (EZBioscience, USA). Each reaction was performed in triplicate. *ACTB* was used as a reference gene to calculate fold-change values using the 2^−ΔΔCt^ method. Primer sequences for RT-qPCR are provided in Supplementary Table S1.

### Western blotting

Cell lysates were prepared using a RIPA buffer (Beyotime, China) with protease inhibitors (MedChemExpress, China) and phosphatase inhibitors (MedChemExpress, China). Cell lysates were then centrifuged at 12,000 rpm for 15 min at 4 °C and supernatants harvested. Protein concentration was measured using a BCA Protein Assay Kit (Thermo, USA) and equal amounts of protein separated by SDS-PAGE, transferred to PVDF membrane (Millipore, USA), and blocked with 5% skim milk or 5% BSA for 1 h at room temperature (RT), followed by overnight incubation with specific primary antibody at 4 °C. The next day, membranes were washed with TBST buffer and incubated with secondary antibody (Proteintech, China) for 1 h at RT. After incubation, membranes were again washed with TBST buffer and visualized using ECL detection reagent (Thermo, USA).

### Co-IP assays

Cells were transfected with a PPP2R2B-FLAG overexpression plasmid or vector control, then harvested with Cell lysis buffer for Western and IP (Beyotime, China) containing 1× Protease Inhibitor Cocktail (MedChemExpress, China) and 1× Phosphatase Inhibitor (MedChemExpress, China). After centrifugation, supernatants were incubated with anti-FLAG magnetic beads (Selleck, China) or Protein A/G magnetic beads (Selleck, China) plus anti-PPP2R2B antibody (ab264160), anti-IPO5 antibody (sc-55,527, Santa) or anti-ISG15 antibody (15981-1-AP, Proteintech) overnight at 4 °C. Bound proteins were eluted in SDS buffer, followed by western blot and MS analyses.

### In vivo animal experiments

All in vivo experimental procedures were ethically compliant and approved by the Institutional Animal Care and Use Committee of Sun Yat-sen University. 4-week-old female BALB/c nude mice were purchased from GemPharmatech (China). To investigate the effects of PPP2R2B in vivo, 3 × 10^6^ T24 and UMUC3 cells stably transfected with PPP2R2B overexpression plasmid or empty vector were injected subcutaneously into the flanks of mice. When the tumors became palpable (approximately 100 mm^3^), the mice were randomly divided into four groups and cisplatin (5 mg/kg) was intraperitoneally administered every 3 days. All mice were sacrificed after 4 weeks, and tumors excised, measured, weighed, and embedded in paraffin for H&E staining and IHC analysis.

To test the efficacy of chaetocin, T24 cells were used to establish subcutaneous xenograft models as described above. Mice were treated with cisplatin (5 mg/kg) or chaetocin (0.5 mg/kg) intraperitoneally every 3 days or daily, respectively, until the endpoint.

### ChIP

ChIP assays were conducted using Magna ChIP A/G (Millipore, USA), according to the manufacturer’s instructions. Briefly, after cell cross-linking and lysis, sonication was applied to shear DNA to 200–1000 bp. Cell lysates were then incubated with protein A/G magnetic beads plus anti-IgG, anti-H3K9me3 (Abcam, ab8898), anti-H3K27me3 (Abcam, ab192985), or anti-SUV39H1 (Sigma, 05-615) antibodies overnight at 4 °C. Finally, pulled down DNA was eluted, purified, and then analyzed by qPCR; the primers used to amplify the purified DNA are listed in Table S1.

### Bioinformatics analysis

*PPP2R2B* mRNA expression profiles in bladder urothelial carcinoma were downloaded from TCGA (https://portal.gdc.cancer.gov/). Data on correlation of *PPP2R2B* mRNA level with clinical prognosis in BC were obtained from GEPIA 2 (http://gepia2.cancer-pku.cn/#index). To explore possible mechanisms involving PPP2R2B in BC pathogenesis and cisplatin resistance, KEGG pathway evaluation and GSEA were conducted using the R (version 4.1.0) package, clusterProfiler (version 4.0.5) [18].

### Statistics

All statistical analyses were performed using GraphPad Prism 9. P values for comparisons between two groups were calculated using the Student’s t test, while one or two-way ANOVA was used to compare values between more than two groups. Differences were considered statistically significant at *P* < 0.05. In this paper, the significance of differences is denoted as follows: *, *P* < 0.05; **, *P* < 0.01; ***, *P* < 0.001; not significant (ns), *P* > 0.05.

## Results

### Identification and characteristics of PPP2R2B as a tumor suppressor in BC

**Fig. 1:**
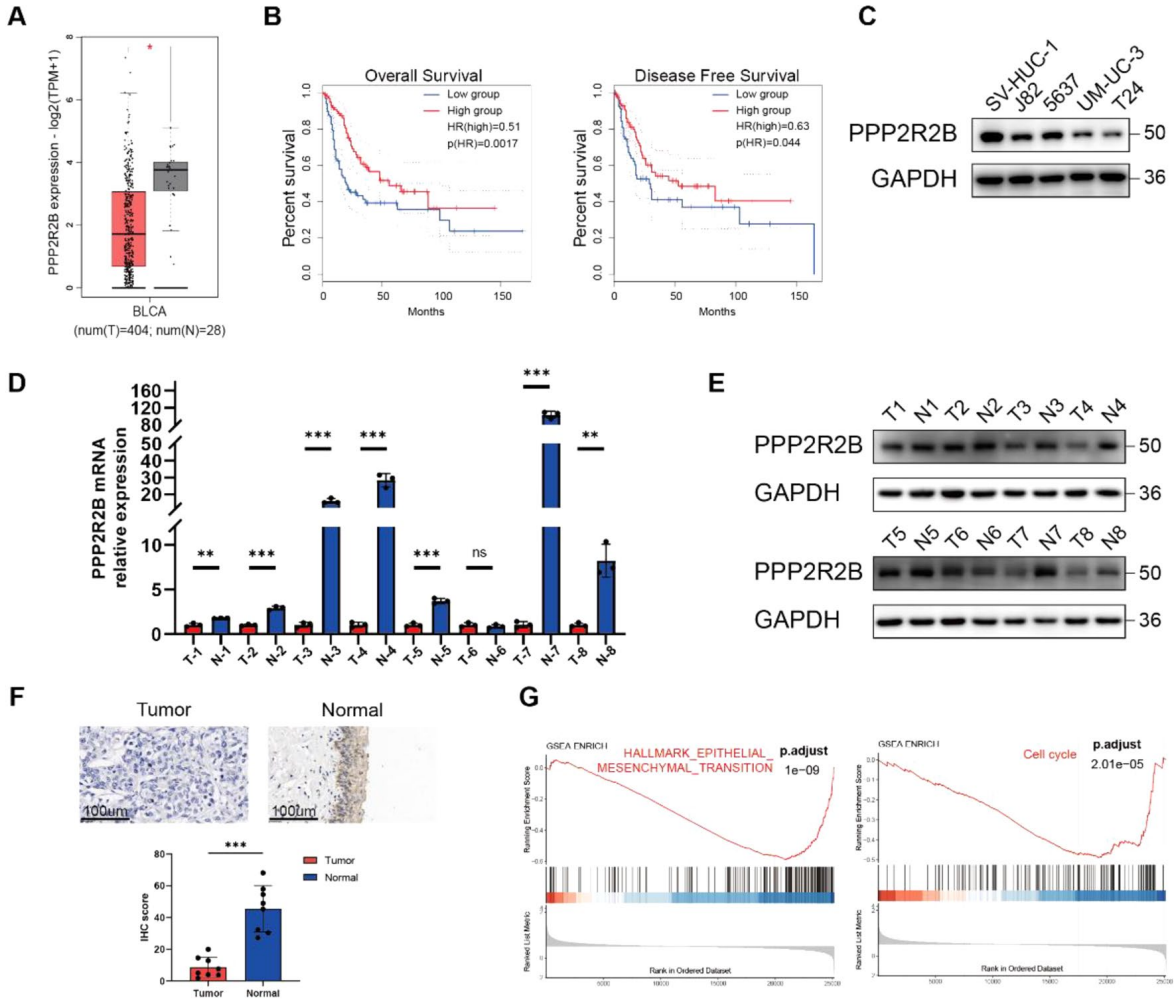
PPP2R2B is identified as a tumor suppressor in bladder cancer (BC). (**A**) Comparison of PPP2R2B expression levels between BC and normal bladder tissue in the Cancer Genome Atlas (TCGA) BC cohort. (**B**) Survival analysis of patients with different PPP2R2B expression levels in TCGA BC cohort. (**C**) Expression of PPP2R2B in an immortalized urothelial cell line (SV-HUC-1) and different BC cell lines. (**D**) Detection of *PPP2R2B* expression levels by RT-qPCR in BC and matched adjacent normal bladder samples (unpaired, 2-tailed t test). (**E**) Detection of PPP2R2B expression levels by western blot in BC and matched adjacent normal bladder samples. (**F**) Detection of PPP2R2B expression levels by IHC in BC and matched adjacent normal bladder samples (unpaired, 2-tailed t test). (**G**) Gene set enrichment analysis enrichment analysis in BC groups with high and low PPP2R2B expression, defined according to median expression in tumors. Three independent experiments were performed. Error bars were represented as mean ± SD

The biological significance of PPP2R2B was first explored using The Cancer Genome Atlas (TCGA) BC dataset. RNA-Seq data from TCGA BLCA cohort revealed that *PPP2R2B* expression was significantly down-regulated in cancer tissues (Fig. 1A). *PPP2R2B* quartile expression level was used as the cut-off value to divide patients into high and low expression groups, and Kaplan–Meier survival analysis showed that patients with low *PPP2R2B* expression had significantly shorter disease-free survival and overall survival (OS) than those with high *PPP2R2B* expression (Fig. 1B).

Then we investigated the differential expression of PPP2R2B in BC cell lines (J82, 5637, UM-UC-3, T24) and an immortalized urothelial cell line (SV-HUC-1) and found that it was expressed at lower levels in BC cell lines than in SV-HUC-1 cells (Fig. 1C). Next, we detected PPP2R2B expression levels in BC and adjacent non-tumor tissues using RT-qPCR, western blot and immunohistochemistry (IHC). In general, PPP2R2B was lower in tumor samples than those in paired adjacent tissue (Fig. 1D-F). Additionally, we performed bioinformatics analysis on TCGA cohort data. Gene set enrichment analysis (GSEA) showed that *PPP2R2B* expression level was closely related to the cell cycle and epithelial-mesenchymal transition (Fig. 1G). Thus, PPP2R2B may play an important role in BC development and have value as a prognostic marker in patients with BC.

### PPP2R2B inhibits the malignant phenotype of BC in vitro

To test our hypothesis that PPP2R2B functions as a tumor suppressor gene, we first performed a series of in vitro experiments. For this purpose, gain and loss of function experiments were conducted in BC cells to determine the role of PPP2R2B (Fig. S1A, B). Silencing or overexpressing PPP2R2B clearly enhanced or inhibited cell growth and colony formation, respectively (Fig. S2A-C). In addition, wound-healing and transwell migration assays showed that PPP2R2B knockdown dramatically promoted cell mobility, whereas PPP2R2B overexpression decreased it (Fig. S2D-G). Moreover, flow cytometry assays were conducted to further explore the potential role of PPP2R2B. Silencing PPP2R2B promoted BC cell transition from G0/1 to S phase, while the reverse was observed with ectopic PPP2R2B expression (Fig. S2H, I). In summary, PPP2R2B may have an important role as a tumor suppressor in BC.

### PPP2R2B mediates cisplatin sensitivity by inhibiting DNA repair in vitro

**Fig. 2:**
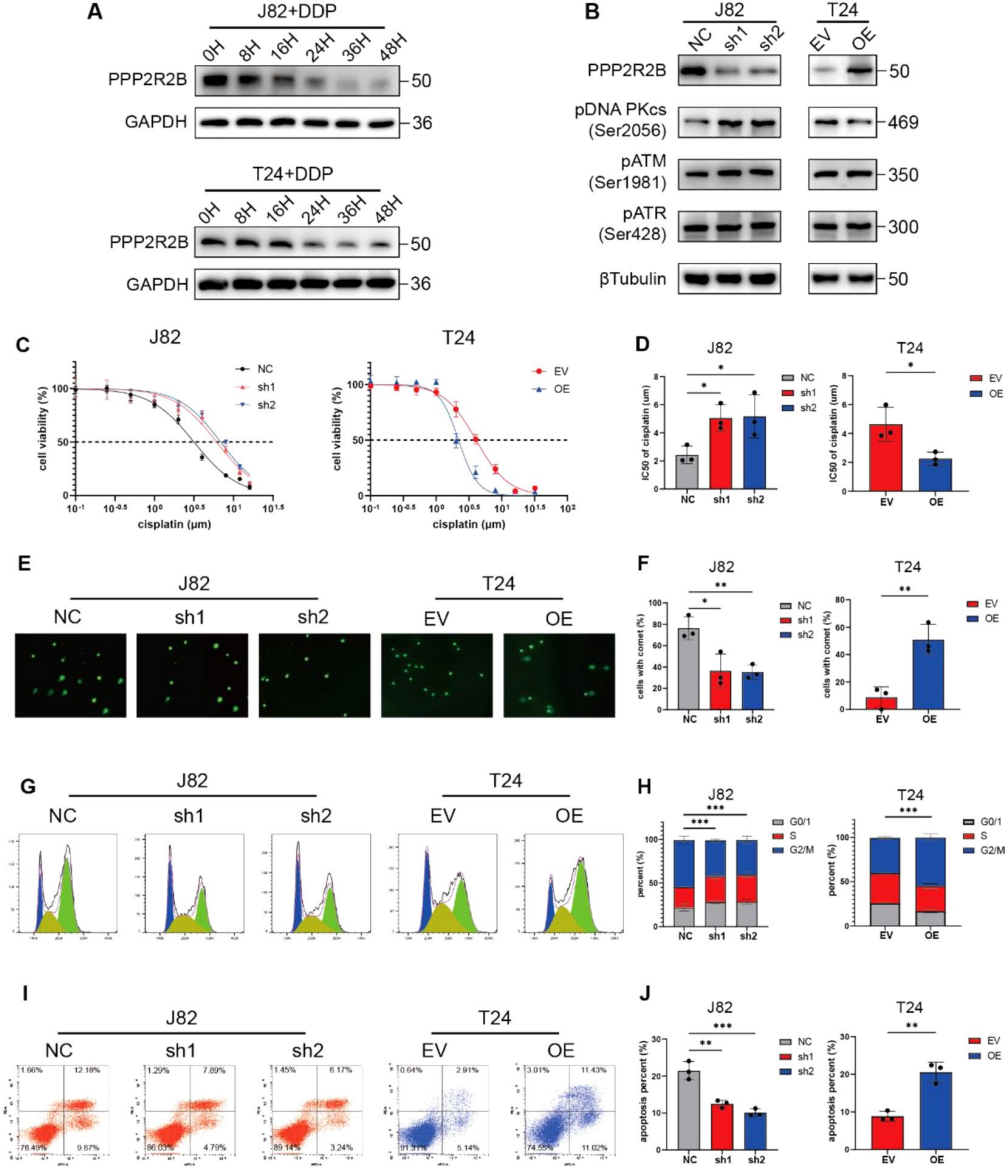
PPP2R2B mediates cisplatin sensitivity by inhibiting DNA repair in vitro. (**A**) Western blot analysis of PPP2R2B levels in J82 and T24 cells treated with cisplatin (DDP). (**B**) Western blot of pDNA PKcs (Ser2056), pATM (Ser1981), and pATR (Ser428) expression in J82 and T24 cells after PPP2R2B knockdown and overexpression. (**C**, **D**) IC_50_ values of cisplatin (DDP) in PPP2R2B-knockdown J82 cells (*n* = 3; one-way ANOVA with Dunnett’s test) and PPP2R2B-overexpression T24 cells (*n* = 3; unpaired, 2-tailed t test). (**E**, **F**) Alkaline comet assays of J82 and T24 cells treated with DDP after PPP2R2B knockdown and overexpression (*n* = 3; one-way ANOVA with Dunnett’s test and unpaired, 2-tailed t test, respectively). (**G**-**J**) Cell cycle (**G**, **H**) (*n* = 3; two-way ANOVA with Tukey’s test for J82 and two-way ANOVA with Bonferroni’s test for T24) and apoptosis (**I**, **J**) (*n* = 3; one-way ANOVA with Dunnett’s test for J82 and unpaired, 2-tailed t test for T24) analysis, indicating the responses of PPP2R2B-silenced J82 cells and PPP2R2B-overexpressing T24 cells to DDP treatment. Three independent experiments were performed. Error bars were represented as mean ± SD

After identifying the functions of PPP2R2B in inhibiting tumor growth and metastasis, further bioinformatics analysis was performed to explore the possible other functions of this protein. Interestingly, GSEA revealed that PPP2R2B was positively related to DNA adduct formation, while negatively related to DNA repair and DNA double-strand break repair (Fig. S3A). As B55 subunits are downregulated in response to irradiation [8], we hypothesized that PPP2R2B is associated with DNA damage repair. Therefore, we first examined the expression of PPP2R2B in response to cisplatin. Treatment with cisplatin decreased PPP2R2B expression in a time-dependent manner (Fig. 2A). Moreover, we found that the activated form of DNA-dependent protein kinase catalytic subunit (DNA PKcs), pDNA PKcs (Ser2056), which participates in non-homologous end joining, increased when PPP2R2B was silenced and decreased when it was overexpressed (Fig. 2B), indicating that PPP2R2B may promote chemosensitivity by regulating DNA repair. We further used a clinical BC cohort that received cisplatin-based chemotherapy to determined our finding [19]. Excitingly, the survival analysis greatly fitted our expectation, in which patients with low PPP2R2B expression had significantly shorter OS than those with high group. Specifically, when median expression level was used as the cut-off value, statistical difference was only shown in the first three years of OS (Fig. S3B, C), while the difference became more significant when using quartile expression level as the cut-off value (Fig. S3D). This reflected the significant effect of PPP2R2B expression level on the efficacy of chemotherapy in bladder cancer.

As a preliminarily investigation of whether PPP2R2B can affect the sensitivity of BC cells to cisplatin, CCK-8 assays were used to determine cisplatin IC_50_ values, and revealed that PPP2R2B knockdown increased the IC_50_ value of cisplatin in BC cells, whereas IC_50_ values were decreased by PPP2R2B overexpression (Fig. 2C, D, Fig. S3E, F). Next, we investigated whether PPP2R2B could regulate DNA damage using comet assays. Cisplatin was applied to induce DNA damage and the level of DNA damage after 24 h was measured using comet assays to detect both single- and double-strand breaks in individual cells [20]. Numbers of comet-positive cells were lower in the PPP2R2B knockdown group than in the control group, while they were higher in the PPP2R2B overexpression group (Fig. 2E, F).

Cisplatin impairs cell cycle progression by inducing arrest in G2/M phase [21, 22]. Therefore, we next evaluated the response of BC cells to 24 h treatment with cisplatin using cell cycle assays. BC cells were arrested in G2/M phase by cisplatin and PPP2R2B knockdown was associated with reduced cisplatin sensitivity, demonstrated by a lower percentage of cells arrested in G2/M phase. Conversely, PPP2R2B overexpression leaded to an increased percentage of cells arrested in G2/M phase (Fig. 2G, H). Flow cytometry analysis was also performed to detect apoptosis following treatment with cisplatin and showed the similar results (Fig. 2I, J). Together, these data suggest that PPP2R2B can enhance BC sensitivity to cisplatin by repressing DNA repair.

### PPP2R2B overexpression sensitized BC cells to cisplatin in vivo

**Fig. 3:**
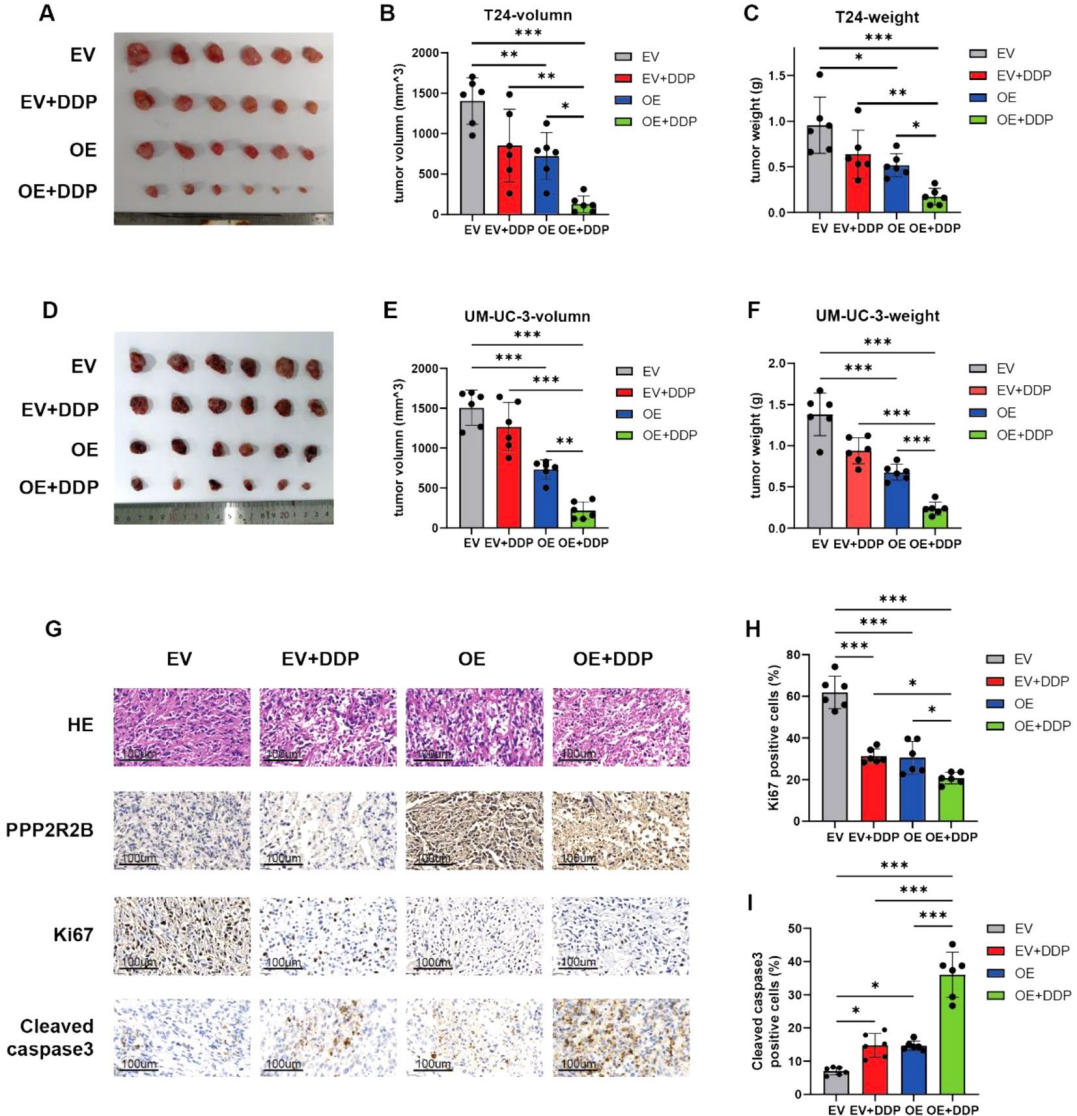
PPP2R2B overexpression sensitizes BC cells to cisplatin in vivo. (**A**–**F**) Images of gross tumors overexpressing PPP2R2B (comprising T24 and UM-UC-3 cells) dissected from subcutaneous xenograft model mice (**A**–**D**). Tumor volume (**B**–**E**) and tumor weight (**C**–**F**), with or without cisplatin (DDP) treatment (*n* = 6; one-way ANOVA with Tukey’s test). (**G**–**I**) H&E staining and IHC analysis of PPP2R2B/Ki67/Cleaved-caspase3 expression performed on xenograft tumors (**G**) and the percentage of positive cells quantified (**H**, **I**) (*n* = 6; one-way ANOVA with Tukey’s test). Error bars were represented as mean ± SD

The potential role of PPP2R2B in vivo was further investigated using a mouse xenograft model treated with or without cisplatin. T24 and UM-UC-3 cells stably transfected with empty vector or PPP2R2B were subcutaneously injected into BALB/c nude mice. When the tumor volume reached approximately 100 mm^3^, mice were randomly distributed to four groups that were intraperitoneally injected with cisplatin or not. Consistent with the data from our in vitro assays, tumor volume and weight were decreased in the PPP2R2B overexpression group compared with the control group (Fig. 3A-F). Meanwhile, PPP2R2B overexpression dramatically enhanced the efficacy of cisplatin in vivo, evidenced by a more prominent reduction in tumor volume and weight than that observed in the empty vector plus cisplatin group (Fig. 3A-F). We then performed IHC analysis of xenograft tumor specimens (Fig. 3G), which revealed that KI67 expression was significantly lower in the PPP2R2B overexpression group (Fig. 3H), whereas levels of cleaved-caspase 3 were higher (Fig. 3I), providing further support for the hypothesis that PPP2R2B inhibits BC cell proliferation and increases their sensitivity to cisplatin.

### PPP2R2B interacts with ISG15 and regulates its transcription

PP2A was previously reported to dephosphorylate DNA PKcs [23]. We treated BC cells with the PP2A-specific inhibitor LB100 and found that pDNA PKcs (S2056) significantly enhanced (Fig. S4A). However, PPP2R2B overexpression still diminished pDNA PKcs (S2056) when PP2A activity was inhibited, and PPP2R2B was unable to interact with it (Fig. S4B, C). These results indicate that the regulation of DNA PKcs by PPP2R2B is largely independent of PP2A.

**Fig. 4:**
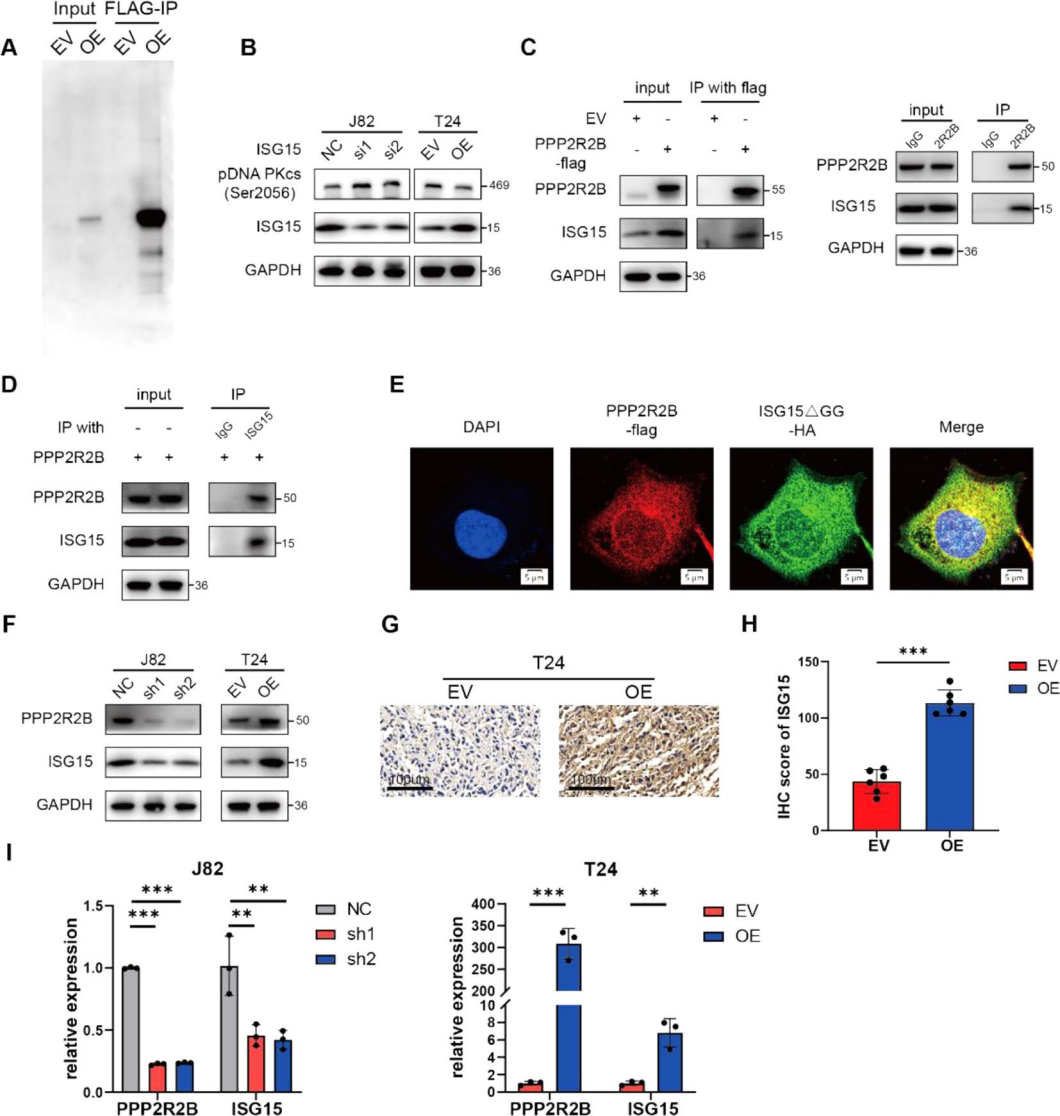
PPP2R2B interacts with ISG15 and regulates its transcription. (**A**) Co-immunoprecipitation was conducted using anti-FLAG magnetic beads. (**B**) Detection of ISG15, pDNA PKcs (Ser2056) and GAPDH by western blot after ISG15 knockdown and overexpression. (**C**, **D**) Western blot to detect the interaction between PPP2R2B and ISG15 after Co-IP. (**E**) Colocalization of PPP2R2B and ISG15 demonstrated by immunofluorescence. (**F**) Detection of PPP2R2B, ISG15 and GAPDH after PPP2R2B knockdown and overexpression. (**G**, **H**) IHC staining of ISG15 performed on xenograft tumors (*n* = 6; unpaired, 2-tailed t test). (**I**) *PPP2R2B* and *ISG15* RNA levels in cells with PPP2R2B silenced (*n* = 3; one-way ANOVA with Dunnett’s test) and overexpressed (*n* = 3; unpaired, 2-tailed t test). Three independent experiments were performed. Error bars were represented as mean ± SD

To explore the mechanism underlying PPP2R2B regulation of BC cisplatin sensitivity, we conducted mass spectrometry (MS) analysis of protein samples purified using anti-FLAG magnetic beads, to identify proteins that physically interacted with PPP2R2B (Fig. 4A); the top 5 proteins are listed in Table 1. We noticed that interferon stimulated gene 15 (ISG15), which has previously been reported to localize at the replication fork, accelerate DNA replication fork progression, and increase chemosensitivity [24–26], were among the top proteins identified in our MS analysis. We therefore hypothesized that ISG15 might be a crucial molecule in PPP2R2B-mediated cisplatin sensitivity. We initially verified that ISG15 inhibited the phosphorylation activation of DNA PKcs and promoted the sensitivity of BC cells to cisplatin (Fig. 4B, Fig. S5A-C). Further, Co-IP was conducted and effectively verified the interaction between PPP2R2B and ISG15 (Fig. 4C, D). As ISG15 is a ubiquitin-like protein which can conjugate and modify other proteins, we used a conjugation-defective form of ISG15 (ISG15ΔGG) in our colocalization assay, to ensure specificity [24, 27]. Similarly, colocalization analysis showed the same result (Fig. 4E).

To determine the role of PPP2R2B on ISG15, PPP2R2B was knocked down and overexpressed to observe the change in ISG15 expression. Western blot showed that PPP2R2B positively regulated ISG15 expression (Fig. 4F). The similar result was also observed on IHC staining of the xenograft tumors above, ISG15 expression was increased in the overexpression group (Fig. 4G, H). Given that PPP2R2B is the regulatory subunit of the PP2A, PPP2R2B may affect the expression of ISG15 by mediating its phosphorylation and we discovered phosphorylation sites of ISG15 in the Eukaryotic Phosphorylation Site Database [28] (Fig. S5D). However, PPP2R2B did not alter the phosphorylation level of ISG15 (Fig. S5E). Meanwhile, overexpression of PPP2R2B had no impact on the degradation of ISG15 when protein translation inhibitor was used (Fig. S5F, G), while PCR revealed the promotion of PPP2R2B to ISG15 transcription (Fig. 4I).

**Table 1:** The top 5 proteins identified by mass spectrometry after Co-IP

Gene symbol	Unique peptide numbers	Unique spectra numbers
PPP2R2B	12	25
PHB1	9	11
MX2	6	7
IPO5	5	5
ISG15	4	5

### PPP2R2B facilitates binding of IPO5 and ISG15 and transports ISG15 into the nucleus

**Fig. 5:**
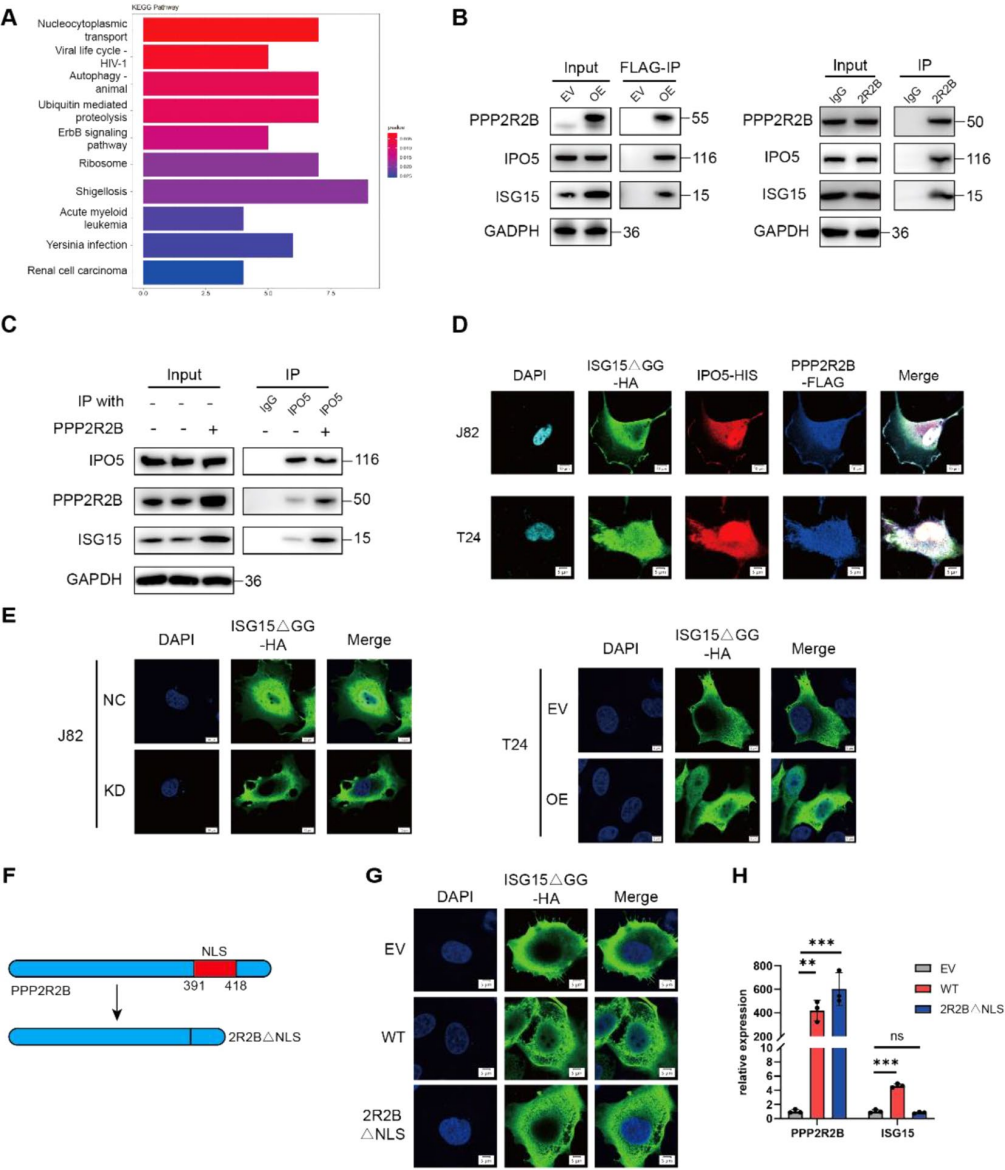
PPP2R2B facilitates binding of IPO5 and ISG15 and transports ISG15 into the nucleus. (**A**) KEGG enrichment analysis of proteins that interacted with PPP2R2B in BC. Nucleocytoplasmic transport was the top label. (**B**) Western blot to detect the interaction between PPP2R2B and IPO5/ISG15 after Co-IP of PPP2R2B. (**C**) Western blot to detected the interaction between IPO5 and PPP2R2B/ISG15 after Co-IP of IPO5. PPP2R2B overexpression enhanced the binding between IPO5 and ISG15. (**D**) Colocalization of PPP2R2B, IPO5, and ISG15 in J82 and T24 cells, demonstrated by immunofluorescence. (**E**) Subcellular localization of ISG15 in PPP2R2B-silenced J82 cells and PPP2R2B-overexpressing T24 cells demonstrated by immunofluorescence. (**F**) Illustration of the NLS-defective PPP2R2B construct (2R2BΔNLS). (**G**) Subcellular localization of ISG15 in control, PPP2R2B and 2R2BΔNLS overexpression group. (**H**) *PPP2R2B* and *ISG15* RNA levels in control, PPP2R2B and 2R2BΔNLS overexpression group. (*n* = 3; one-way ANOVA with Dunnett’s test). Three independent experiments were performed. Error bars were represented as mean ± SD

The phenomenon that PPP2R2B interacts directly with ISG15 but affects its transcription is puzzling. It is noteworthy that ISG15 is located at replication forks and its function may be correlated with its subcellular distribution. Interestingly, we conducted Kyoto Encyclopedia of Genes and Genomes (KEGG) enrichment analysis on the proteins identified by MS and found that nucleocytoplasmic transport was the most significant label in the enrichment results (Fig. 5A), which indicated the key role of PPP2R2B in assisting nucleocytoplasmic transport. To test this hypothesis, we first predicted whether PPP2R2B contains a nuclear localization sequence (NLS) using cNLS Mapper [29, 30]. There was an NLS sequence located between amino acids 391 and 418 of PPP2R2B (Fig. S6A), indicating that the protein has potential for nuclear import. Moreover, we noticed that importin 5 (IPO5), a nuclear import protein [31, 32], was one of the leading proteins detected in our MS analysis (Table 1). Therefore, we hypothesized that PPP2R2B may facilitate the binding of IPO5 and ISG15 and then transport ISG15 into the nucleus.

The interaction of PPP2R2B with IPO5 and ISG15 was initially confirmed by western blot (Fig. 5B), and we demonstrated that PPP2R2B overexpression could increase the binding between IPO5 and ISG15 (Fig. 5C). Further, we conducted colocalization analysis to visualize these interactions. Consistent with the findings from our previous experiments, immunofluorescence staining demonstrated the colocalization of PPP2R2B, IPO5, and ISG15 in the J82 and T24 cell lines (Fig. 5D). In subsequent experiments, we explored the change in the nuclear distribution of ISG15 mediated by PPP2R2B. Immunofluorescence staining showed that PPP2R2B knockdown decreased ISG15 nuclear distribution, which was increased on PPP2R2B overexpression (Fig. 5E). Besides, ISG15 expression in subcellular fractions was also detected by western blot and the same phenomenon was observed (Fig. S6B).

NLS-defective form of PPP2R2B (2R2BΔNLS) was constructed to further validate its nucleocytoplasmic transport function (Fig. 5F). Co-IP showed that the capacity of PPP2R2B to bind IPO5 was lost following deletion of the NLS sequence (Fig. S6C). Moreover, 2R2BΔNLS is incapable of promoting the nuclear translocation of ISG15 compared to the wild type (Fig. 5G). Meanwhile, PCR also found that ISG15 expression could not be promoted by 2R2BΔNLS (Fig. 5H). These results suggest that PPP2R2B promotes the nuclear translocation of ISG15, which is essential to promote the expression of ISG15.

### ISG15 is the core molecule in PPP2R2B-regulated sensitivity to cisplatin

**Fig. 6:**
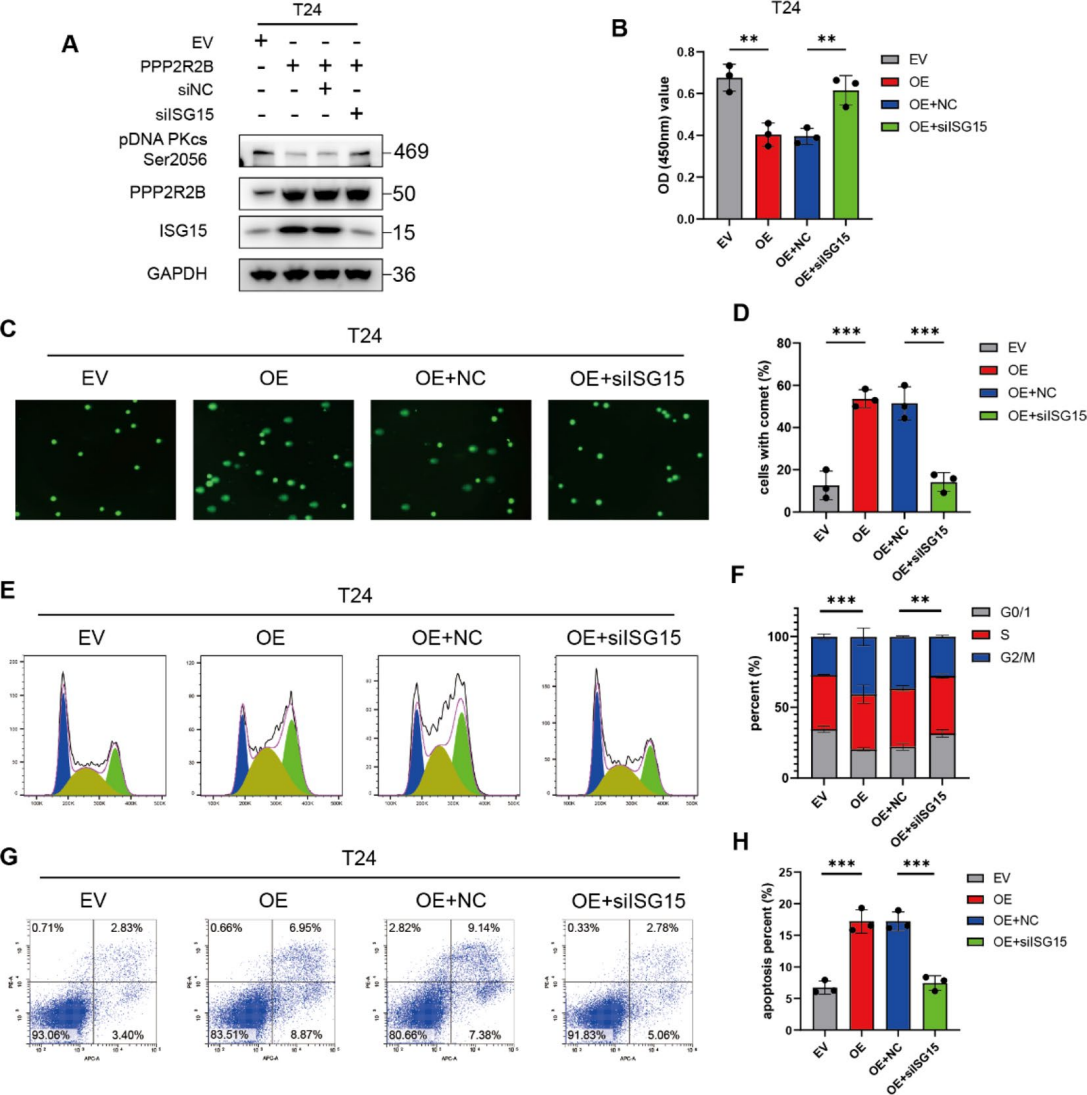
ISG15 is a core molecule in PPP2R2B-regulated sensitivity to cisplatin. (**A**) Silencing ISG15 rescued PPP2R2B overexpression-induced down-regulation of pDNA PKcs (Ser2056). (**B**–**H**) Cell viability (**B**) (*n* = 3, one-way ANOVA with Tukey’s test), comet (**C**, **D**) (*n* = 3, one-way ANOVA with Tukey’s test), cell cycle (**E**, **F**) (*n* = 3, two-way ANOVA with Tukey’s test), and apoptosis (**G**, **H**) (*n* = 3, one-way ANOVA with Tukey’s test) assays to evaluate the effect of ISG15 depletion on PPP2R2B-overexpression in T24 cells treated with cisplatin. Three independent experiments were performed. Error bars were represented as mean ± SD

To test whether PPP2R2B plays a major role in regulating cisplatin sensitivity through ISG15, we knocked down ISG15 in cells with PPP2R2B overexpressed. ISG15 depletion rescued the downregulation of pDNA PKcs (S2056) induced by PPP2R2B overexpression (Fig. 6A). Further, CCK-8 assays demonstrated that ISG15 knockdown abolished PPP2R2B overexpression-induced chemosensitivity (Fig. 6B). Moreover, knockdown of ISG15 in PPP2R2B-overexpression cells resulted in significantly lower numbers of cells with comet tails (Fig. 6C, D), indicating a decrease in DNA breaks, and implying that silenced ISG15 enhances the chemoresistance of BC cells with PPP2R2B overexpressed. Cell cycle and apoptosis assays also showed that ISG15 depletion decreased G2/M arrest (Fig. 6E, F) and cell death (Fig. 6G, H) induced by cisplatin in BC cells with PPP2R2B overexpressed. Conversely, the effect of PPP2R2B knockdown on sensitization to cisplatin was reversed by ISG15 overexpression (Fig. S7A-H). These findings support a potential role for ISG15 in mediating the chemo-sensitizing effect of PPP2R2B.

### PPP2R2B enhances ISG15 transcription by activating the STING pathway

**Fig. 7:**
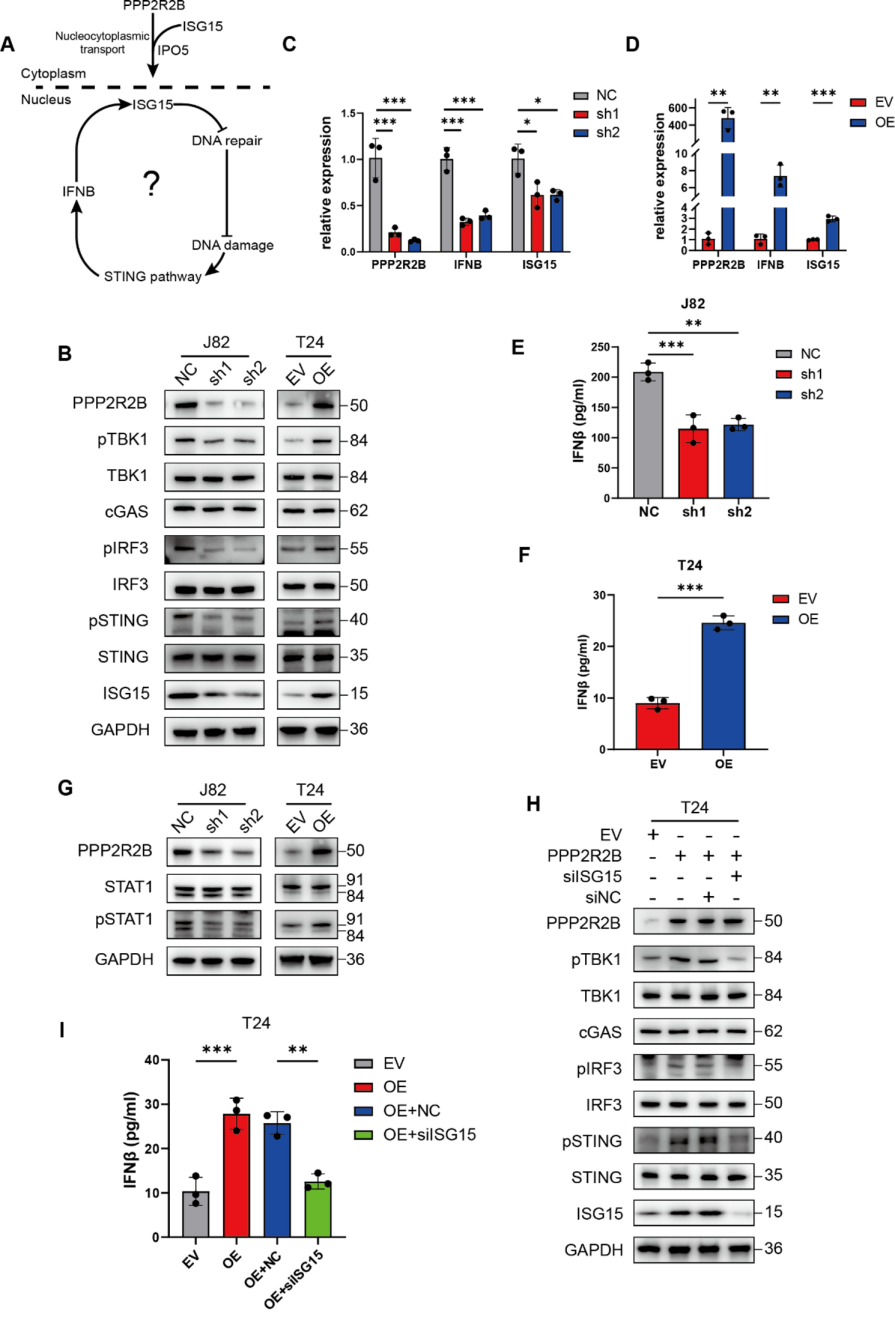
PPP2R2B enhances ISG15 transcription by activating the STING pathway. (**A**) The possible mechanism by which PPP2R2B increases ISG15 expression. (**B**) Western blot of STING pathway proteins after PPP2R2B knockdown and overexpression. (**C**, **D**) *ISG15* and *IFNB* RNA levels in cells with PPP2R2B silenced (*n* = 3; one-way ANOVA with Dunnett’s test) and overexpressed (*n* = 3; unpaired, 2-tailed t test). (**E**, **F**) ELISA showing IFNB concentration in J82 (*n* = 3; one-way ANOVA with Dunnett’s test) and T24 (*n* = 3; unpaired, 2-tailed t test) cells after PPP2R2B knockdown and overexpression. (**G**) Detection of PPP2R2B, STAT1, pSTAT1, and GAPDH by western blot after PPP2R2B knockdown and overexpression. (**H**) Silencing ISG15 reversed PPP2R2B overexpression-induced activation of the STING pathway in T24 cells. (**I**) ISG15 depletion diminished PPP2R2B overexpression-induced IFNB production detected by ELISA (*n* = 3; one-way ANOVA with Tukey’s test). Three independent experiments were performed. Error bars were represented as mean ± SD

The relationship between the nuclear translocation of ISG15 and its increased expression remains unclear in the above experiments. ISG15 is an interferon-stimulated gene, which is induced by DNA damage and activation of the STING pathway, and has been reported to have a positive feedback effect on IFNB [33, 34]. Hence, PPP2R2B may induce STING pathway activation and interferon expression by promoting ISG15 nuclear translocation and inhibiting DNA repair, resulting in increased ISG15 (Fig. 7A). First, we investigated the effects of PPP2R2B on the STING pathway and IFNB, since STING pathway activation is crucial for ISG15 expression. When PPP2R2B was depleted, TBK1, IRF3 and STING phosphorylation was decreased, indicating inactivation of the STING pathway. Conversely, PPP2R2B overexpression activated the STING pathway (Fig. 7B). Similar changes were observed in *IFNB* and *ISG15* mRNA expression (Fig. 7C, D). What’s more, ELISA was performed to detect IFNB, which showed positive regulation of IFNB by PPP2R2B (Fig. 7E, F). As ISG15 expression is induced by type I IFN via signal transducer and activator of transcription (STAT) family proteins [34, 35], we also analyzed STAT1 phosphorylation levels by western blot. PPP2R2B knockdown reduced pSTAT1 levels and the opposite effect was observed in cells overexpressing PPP2R2B (Fig. 7G).

To further confirm our conclusion, we depleted ISG15 in T24 cells overexpressing PPP2R2B. ISG15 knockdown reversed PPP2R2B overexpression-induced activation of the STING pathway and the IFNB production (Fig. 7H, I). Consistent with above findings, we found a positive correlation between PPP2R2B/ISG15/pSTING in IHC staining of clinical samples (Fig. S8A, B). These findings suggest that PPP2R2B-induced nuclear import of ISG15 inhibits DNA repair and promotes its own expression through activation of the STING pathway.

### SUV39H1-mediated histone 3 lysine 9 trimethylation (H3K9me3) repressed PPP2R2B expression

**Fig. 8:**
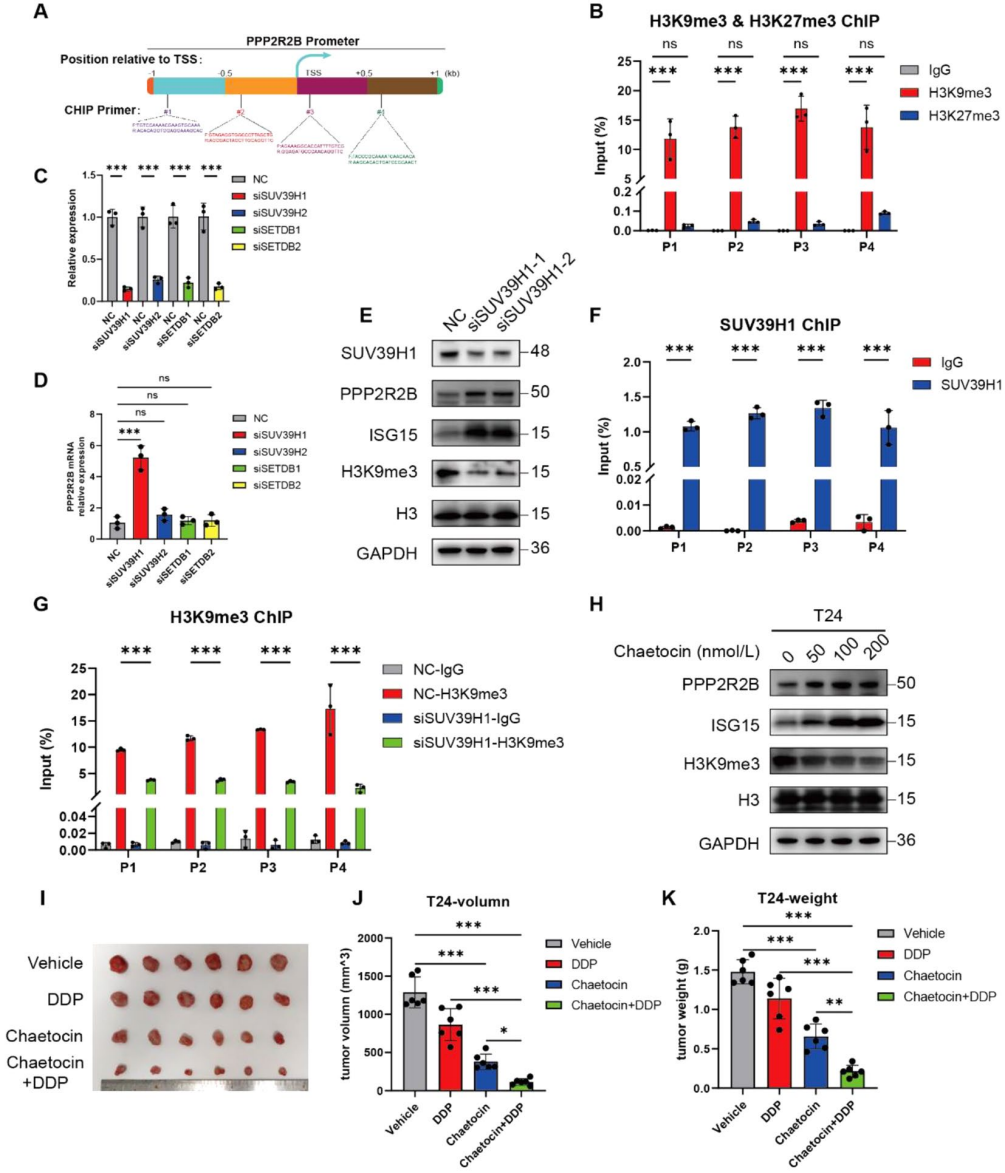
SUV39H1-mediated H3K9me3 represses PPP2R2B expression. (**A**) Schematic diagram showing ChIP primer locations across the *PPP2R2B* promoter region. (**B**) ChIP-qPCR of H3K9me3- and H3K27me3-enriched samples at the *PPP2R2B* promoter in T24 cells using the primers shown in (A) (*n* = 3; two-way ANOVA with Dunnett’s test). (**C**, **D**) *PPP2R2B* RNA levels (*n* = 3; one-way ANOVA with Dunnett’s test) in cells with H3K9me3-related histone methyltransferases (SUV39H1/2 and SETDB1/2) silenced (*n* = 3; unpaired, 2-tailed t test). (**E**) Detection of SUV39H1, PPP2R2B, ISG15, H3K9me3, H3, and GAPDH by western blot in T24 cells after SUV39H1 knockdown. (**F**, **G**) ChIP-qPCR of SUV39H1 (F) (*n* = 3; two-way ANOVA with Bonferroni’s test) and H3K9me3 (**G**) (*n* = 3; two-way ANOVA with Tukey’s test) -enriched samples at the *PPP2R2B* promoter in T24 cells using the primers shown in (**A**). (**H**) Detection of PPP2R2B, ISG15, H3K9me3, H3, and GAPDH by western blot in T24 cells after treatment with the specific SUV39H1 inhibitor, chaetocin. (**I**) Images of gross tumors dissected from subcutaneous xenograft model mice treated with different strategies. (**J**, **K**) Tumor volume (**J**) and tumor weight (**K**) following different treatments (*n* = 6; one-way ANOVA with Tukey’s test). Three independent experiments were performed. Error bars were represented as mean ± SD

Finally, as PPP2R2B is a tumor suppressor, which is difficult to overexpress as a possible therapeutic target, we searched for an upstream mechanism that negatively regulates PPP2R2B. Few PPP2R2B mutations are present in TCGA data (Fig. S9A) and it was previously reported that low PPP2R2B expression is caused by promoter DNA hypermethylation and histone hypermethylation [4, 7]. Therefore, we investigated *PPP2R2B* promoter DNA methylation levels using TCGA data and found that it was not hypermethylated in BC (Fig. S9B), indicating that the low PPP2R2B expression may be caused by histone methylation. H3K9me3 and H3K27me3 are the two most commonly studied sites of histone methylation that mediate gene silencing [36, 37], and related histone methyltransferases, including SUV39H1/2, SETDB1, and EZH2, are highly expressed in BC (Fig. S9C-F). Therefore, we conducted ChIP assays to investigate these two types of histone methylation. ChIP assay primers were designed at 0.5 kb intervals around the transcriptional start site with reference to a previous study (Fig. 8A) [7]. Interestingly, ChIP assay results revealed that H3K9me3 was highly enriched in the promoter region of *PPP2R2B* compared with H3K27me3 and a negative control, and the enrichment in BC cells were higher than SV-HUC-1, indicating a key role for H3K9me3 in *PPP2R2B* expression regulation (Fig. 8B, Fig. S10A).

To explore the specific molecular events that induced PPP2R2B silencing, we depleted the major genes that mediate H3K9me3, including SUV39H1/2 and SETDB1/2 (Fig. 8C). SUV39H1 knockdown significantly increased *PPP2R2B* mRNA expression (Fig. 8D), while western blot analysis revealed upregulation of PPP2R2B and ISG15 following the decrease in H3K9me3 when SUV39H1 was silenced (Fig. 8E). In the clinical BC samples, we also found a negative correlation between SUV39H1/PPP2R2B (Fig. S10B), which supports the role of SUV39H1 in the regulation of PPP2R2B. Moreover, further evidence of SUV39H1-mediated PPP2R2B repression was provided by ChIP assays. SUV39H1 was significantly enriched at the *PPP2R2B* promoter region and the enrichment in BC cells were higher than SV-HUC-1 (Fig. 8F, Fig. S10C), while H3K9me3 modification at the *PPP2R2B* promoter region was decreased after SUV39H1 knockdown (Fig. 8G).

Chaetocin is a specific inhibitor of SUV39H1 both in vitro and in vivo and plays an effective anti-tumor role in various malignancies [38–43]. Given our finding that SUV39H1 negatively regulates PPP2R2B, we hypothesized that chaetocin treatment would increase PPP2R2B expression. Indeed, western blot analysis showed that chaetocin increased PPP2R2B expression in a dose-dependent manner (Fig. 8H). In vivo xenograft experiments revealed that chaetocin dramatically inhibited tumor growth and that the inhibitory effect was even more pronounced when it was combined with cisplatin (Fig. 8I-K). In addition, increases in PPP2R2B, ISG15 and pSTING in chaetocin-treated group were also observed by IHC staining (Fig. S10D). In summary, as a SUV39H1-specific inhibitor, chaetocin may suppress tumor growth and increase the sensitivity of BC cells to cisplatin by up-regulating PPP2R2B expression.

## Discussion

**Fig. 9:**
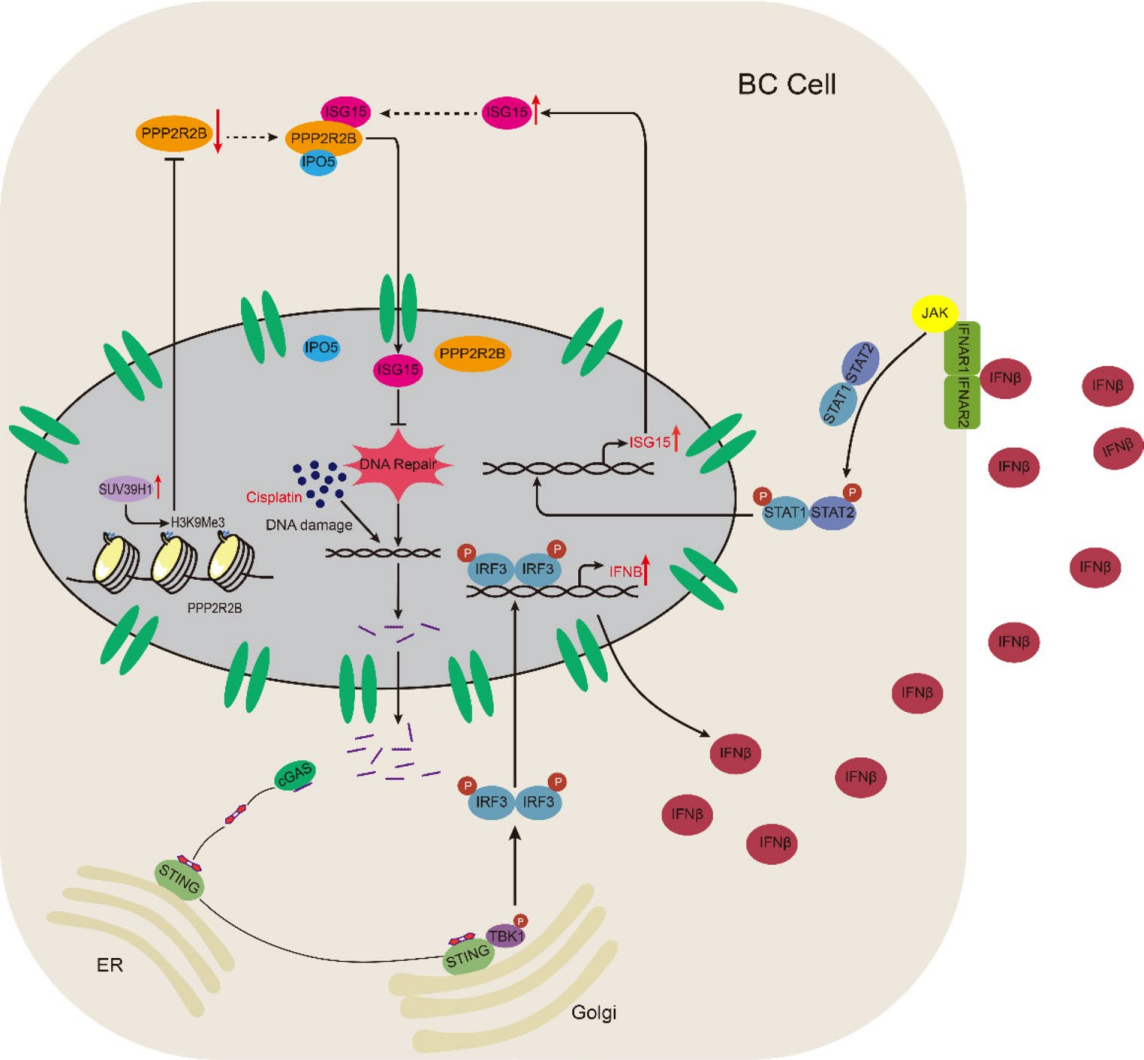
Schematic diagram shows the mechanism that PPP2R2B mediates cisplatin sensitivity. PPP2R2B, which is down-regulated by SUV39H1-mediated trimethylation of H3K9, promotes entry of ISG15 into the nucleus by mediating the binding of IPO5 and ISG15. Nuclear translocation of ISG15 inhibited DNA repair, further increasing ISG15 expression through activation of the STING pathway

Withstanding DNA damage is an important mechanism of cisplatin resistance and treatments that exploit defects in DNA damage repair have been implemented for many cancers [44–46]. In the present study, we found that low PPP2R2B expression was correlated with cisplatin resistance. In terms of mechanism, we found a novel function of PPP2R2B as a nucleocytoplasmic transport molecule. PPP2R2B promoted ISG15 entry into the nucleus by mediating binding of IPO5 with ISG15. DNA repair was inhibited by the nuclear translocation of ISG15, which activated the STING pathway and further enhanced ISG15 expression. Besides, PPP2R2B was down-regulated by SUV39H1-mediated trimethylation of H3K9 in its promoter region, while the SUV39H1 specific inhibitor, chaetocin, could restore PPP2R2B expression to facilitate effective cisplatin therapy (Fig. 9). These data indicate that PPP2R2B may serve as a reliable biomarker to predict the clinical outcomes of patients with BC treated using cisplatin and that SUV39H1 inhibitor is a potential strategy to overcome cisplatin resistance.

PPP2R2B is a regulatory subunit of a PP2A heterotrimeric complex [47], which has a role in recognizing dephosphorylated substrates, including cyclin E1 and PDK1, which participate in the cell cycle and tumor proliferation [4, 48]. Decreased PPP2R2B expression in response to radiation is accompanied by increased expression of DNA repair genes, suggesting a relationship between PPP2R2B and DNA repair [8]; however, a detailed understanding of this process and of the specific function of PPP2R2B remain limited. In the present study, for the first time, we detected PPP2R2B-specific binding proteins in BC by IP-MS. Unexpectedly, the proteins identified by IP-MS were mainly enriched for nucleocytoplasmic transport function, rather than dephosphorylation, and an NLS sequence was identified in PPP2R2B, which had not previously been reported, further arousing our interest. We also discovered that PPP2R2B mediates the binding of IPO5 and ISG15 and promotes transport of the latter into the nucleus. This novel function of PPP2R2B as a molecule that assists in nucleocytoplasmic transport indicates that it has diverse functions and provides researchers with a new perspective.

ISG15 is a ubiquitin-like protein stimulated by type I interferon, which can exist either as a free molecule or covalently conjugated to target proteins via a C-terminal LRLRGG motif, which is referred to as ISGylation [49, 50]. ISG15 and ISGylation may regulate a wide range of cell functions and processes in response to cellular stress [51]. Although some studies have demonstrated that ISG15 accelerates DNA replication fork progression and increases chemosensitivity, its roles in chemotherapy resistance remain controversial [24, 26, 52]. In our study, we found that ISG15 could inhibit DNA PKcs activation, thereby increasing the susceptibility of BC cells to cisplatin. Interestingly, DNA repair inhibition further enhanced ISG15 expression through activation of the STING pathway, consistent with previous research revealing that ISG15 positively regulates type I IFN signaling [34]; however, the previous study did not describe the relationship between ISG15 and activation of the STING pathway, our results fill this gap.

Histone modifications and the enzymes that implement them have crucial roles in regulating gene expression [53], and these processes are often dysregulated in human cancers, thus disrupting gene expression balance [54]. Reversible and precise histone lysine methyltransferase (KMT) regulation over time and space is essential for epigenome homeostasis. In recent years, KMT dysregulation has been associated with tumor development, metastasis, and chemoresistance, as well as the immune microenvironment, while KMT inhibitors are currently being applied in multiple preclinical and clinical trials and have shown promising results in a variety of malignancies [55]. As PPP2R2B is a tumor suppressor, upstream activation of its expression is feasible. PPP2R2B silencing is reported to be due to promoter DNA methylation in colorectal cancer and trimethylation of H3K27 in breast cancer [4, 7]. Moreover, EZH2 inhibitors can activate PPP2R2B expression, thus increasing breast cancer sensitivity to anti-HER2 therapy [7]. In this study, we found that H3K9 trimethylation was the main cause of PPP2R2B silencing in BC, and that the SUV39H1 inhibitor, chaetocin, significantly increased PPP2R2B expression and the sensitivity of BC cells to cisplatin. These findings strongly suggest that PPP2R2B regulation is tissue and organ-specific.

In summary, our study reveals a novel function of PPP2R2B in nucleocytoplasmic transport. PPP2R2B not only plays an important role in inhibiting growth and metastasis of BC, but also increases the sensitivity of BC tumors to cisplatin. These data indicate the potential potence of PPP2R2B as a biomarker for prognosis and cisplatin response in patients with BC. In addition, combination treatment with chaetocin and cisplatin may be an effective strategy to overcome chemoresistance in patients with BC.

### Electronic supplementary material

Below is the link to the electronic supplementary material.

Supplementary Material 1

Supplementary Material 2

## Data Availability

The data used in the current study are available from the corresponding authors upon reasonable request.

## Abbreviations

BC: Bladder cancer
PP2A: Serine/threonine protein phosphatase 2 A
Co-IP: Co-immunoprecipitation
ChIP: Chromatin immunoprecipitation
TCGA: The Cancer Genome Atlas
OS: Overall survival
GSEA: Gene set enrichment analysis
DDP: Cisplatin
MS: Mass spectrometry
KEGG: Kyoto Encyclopedia of Genes and Genomes
NLS: Nuclear localization sequence
KMT: Histone lysine methyltransferase
