# Supplementary material for: Nuclear translocation of ISG15 regulated by PPP2R2B inhibits cisplatin resistance of bladder cancer
Source: Cell Mol Life Sci. 2024 Jul 8;81(1):292. doi: 10.1007/s00018-024-05320-1 (PMC11335216; doi:10.1007/s00018-024-05320-1)
Supplement: Supplementary file 1 — Supplementary Material 1 [file 18_2024_5320_MOESM1_ESM.docx]

Supplemental Information for

**Nuclear Translocation of ISG15 Regulated by PPP2R2B Inhibits Cisplatin Resistance of Bladder Cancer**

Gaowei Huang^1,2*^, Jinwen Liu^1*^, Anze Yu^1*^, Chenggong Luo^3*^, Jiangquan Zhu^1^, Yinghan Wang^1^, Ziran Dai^1^, Lizhen Zhang^1^, Zihao Feng^1^, Jun Lu^1^, Zhong Dong^4#^, Junhang Luo^1#^, Wei Chen^1#^, Zhenhua Chen^1#^.

**Affiliations:**

1. Department of Urology, The First Affiliated Hospital, Sun Yat-sen University, Guangzhou 510080, Guangdong, China.
2. Department of Urology, Shenzhen People's Hospital (The Second Clinical Medical College, Jinan University, The First Affiliated Hospital, Southern University of Science and Technology), Shenzhen, China.
3. Department of Urology, Guizhou Provincial People's Hospital, Guizhou University, Guiyang 550002, Guizhou, China.
4. Department of Urology, Huizhou Central people's Hospital, Huizhou, Guangdong, 516001, China.

* These authors contribute equally to this work: Gaowei Huang, Jinwen Liu, Anze Yu, Chenggong Luo

# **Corresponding authors:**

Zhenhua Chen ([chenzhh75@mail.sysu.edu.cn](mailto:chenzhh75@mail.sysu.edu.cn)), Wei Chen ([chenw3@mail.sysu.edu.cn](mailto:chenw3@mail.sysu.edu.cn)), Junhang Luo ([luojunh@mail.sysu.edu.cn](mailto:luojunh@mail.sysu.edu.cn)) and Zhong Dong ([hzdongzhong@126.com](mailto:hzdongzhong@126.com))

**This file includes:**

Supplemental methods

Fig. S1 to S10

Table S1

**Supplemental methods**

**Plasmids and siRNAs transfection**

PPP2R2B, ISG15, ISG15ΔGG, and IPO5 overexpression plasmids, with or without corresponding tags were obtained from Genecreate Biotech Co. (Wuhan, China). The viral packaging plasmids, pMD2.G and psPAX2, as well as shRNA of PPP2R2B were obtained from Tsingke Biotech Co (Beijing, China). siRNAs were purchased from Ribo Biotech Co. (Guangzhou, China); siRNA and shRNA sequences are provided in Supplementary Table S1. Polyethylenimine transfection reagent (Polysciences, USA) was used to transfect the PPP2R2B knockdown and overexpression plasmids, pMD2.G and psPAX2, into HEK-293T cells. After transfection for 48 h, medium containing viral particles was harvested and cells then transduced using 10 µg/ml polybrene (YEASEN, China). Infected cells were screened using 2 µg/ml puromycin 48 h later. Jetprime (Polyplus, France) was used for transfection of plasmids and siRNAs in tumor cell lines, according to the manufacturer’s instructions.

**CCK-8 and colony formation assays**

Cell proliferation assays were carried out using CCK-8 reagent (MedChemExpress, China). Cells (1000 per well) were cultured in 96-well plates in 100 μl of medium containing 10% FBS, which was replaced with medium containing 10% CCK-8 reagent, cultured at 37℃ without light, and optical absorbance at 450 nm detected 1 h later. To determine IC_50_ values of cisplatin, 3000 cells per well were plated in 96-well plates and gradient concentrations of cisplatin added the next day. After 48 h treatment, optical absorbance was detected as described above, and the IC_50_ value of cisplatin calculated.

For colony formation assays, 500 cells per well were seeded in 6-well plates and cultured for 2 weeks. Colonies were fixed with 4% paraformaldehyde and then stained with 0.1% crystal violet. Finally, numbers of colonies containing more than 50 cells were counted.

**Wound-healing and transwell migration assays**

To perform wound-healing assays, a 200 µL pipette tip was used to scratch BC cells cultured in dishes. Movement of BC cells into the artificial scratches was then measured as the distance of wound closure. Transwell migration assays were performed using a 24-well transwell chambers (Corning, USA). Approximately 50,000 cells were resuspended in serum-free medium and seeded into the upper chambers. Then, medium containing 10% FBS was added to the lower chambers as a chemoattractant. Cells crossing the membrane were fixed, stained, and counted.

**Flow cytometry**

Cells were fixed in 70% alcohol overnight at 4°C after treatment, then washed three times with PBS, and stained with a mixture of RNase A and propidium iodide for 30 min at 37℃. Stained cells were then subjected to flow cytometry. FlowJo software was used to conduct cell cycle analysis. An AnnexinV Alexa Fluor647/PI Apoptosis Detection Kit (4Abio, China) was used to assess apoptosis, according to the manufacturer’s instructions.

**Comet assays**

DNA double-strand breaks were evaluated using a Single Cell Gel Electrophoresis Assay kit (Trevigen, USA), following the manufacturer’s instructions. After electrophoresis, slides were air-dried and then stained with SYBR Gold (Thermo, USA) for 30 min at RT. Finally, slides were rinsed in water, completely dried at 37°C, images recorded by inverted fluorescence microscopy, and the percentage of positive cells with a tail calculated.

**I****mmunofluorescence and IHC assays**

PPP2R2B-FLAG, ISG15ΔGG-HA, and IPO5-HIS were transfected into cells to examine the subcellular localization of the proteins. Cells were plated onto confocal dishes, fixed with 4% paraformaldehyde and permeabilized with 0.5% Triton X-100 for 15 min, then incubated in PBS containing 0.05% Tween-20 and 5% BSA for 1 h, followed by incubation overnight at 4°C with FLAG (F1804, Sigma) and HA (51064-2-AP, Proteintech) -specific primary antibodies. Next, cells were washed and incubated with CoraLite488- and Alexa Fluor 647-conjugated secondary antibodies for 1 h. After secondary antibodies were washed, cells were incubated with iFluor 555-conjugated HIS-specific primary antibody (A01801, Genscript) overnight at 4°C, washed three times, and DAPI used to stain cell nuclei before examination under a confocal microscope.

Paraffin-embedded tissue sections were baked in an oven at 65°C for 2 h, then dewaxed, rehydrated, and antigen retrieval conducted using antigen retrieval solution. Subsequently, sections were treated with 3% H_2_O_2_ to block endogenous peroxidase activity, followed by incubation with 5% BSA. Sections were then incubated overnight with anti-PPP2R2B, Ki67, cleaved-caspase-3, or ISG15 antibodies at 4°C, then incubated with HRP-conjugated secondary antibody (ZSGB-BIO, China) for 30 min at RT. DAB substrate solution (ZSGB-BIO, China) was used for visualization, followed by counterstaining with hematoxylin, dehydration, and mounting.

**Nuclear and cytoplasmic fractionation**

Cells were digested, harvested, and then washed three times with PBS. Then, Nuclear and Cytoplasmic Extraction Reagents (Thermo, USA) were used in accordance with the manufacturer's instructions. Nuclear and cytosolic fraction samples were stored at -80°C before western blot analysis.

**ELISA**

Culture medium supernatants from BC cells were harvested, concentrated, and stored at -80°C before detection. Levels of IFNB in supernatants were detected using a Human IFNB ELISA Kit (Solarbio, China), following the manufacturer’s protocol. A standard curve was constructed based on the absorbance of the standard protein and used to calculate the concentration of IFNB.

**Mass spectrometry**

The samples from Co-IP were performed enzymatic digestion then peptides were extracted. Further, the peptides were enriched in trap column and desalted, and then entered a self-packed C18 column and separated at a flow rate of 300nL/min using Thermo UltiMate 3000 UHPLC. The peptides separated by liquid phase chromatography were ionized by a nanoESI source, and then passed to a tandem mass spectrometer Q-Exactive HF X (Thermo, USA) for detection. Finally, the protein identification software was used to identify the proteins through UniProt protein database.

Fig. S1

**
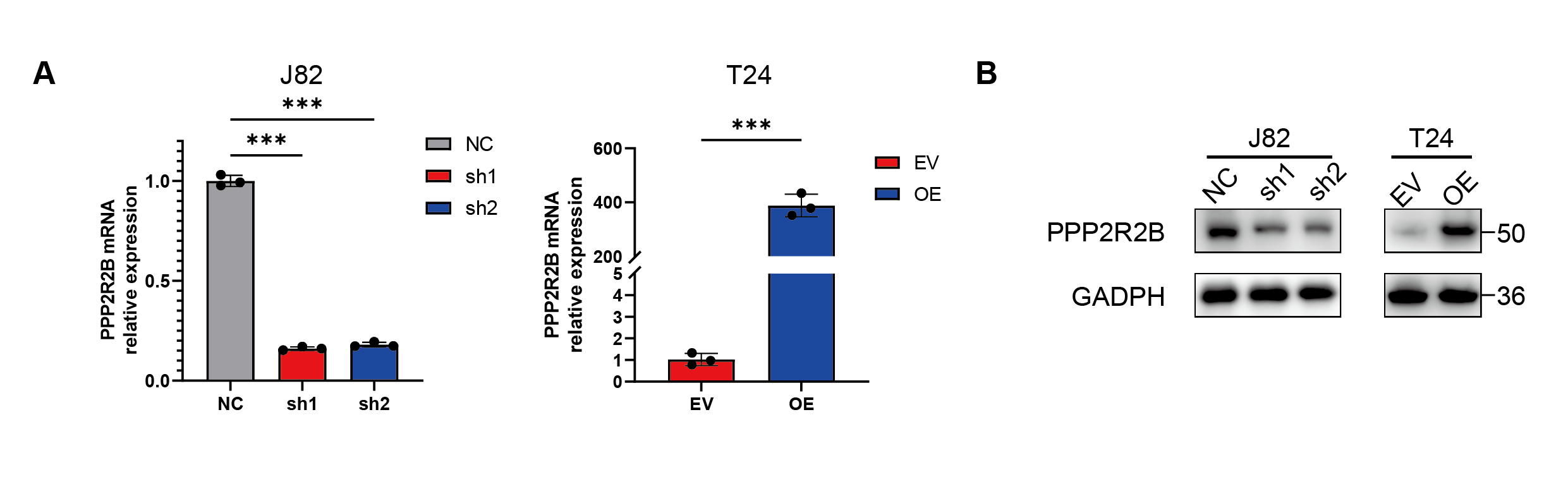
 Fig. S1** **Identification of PPP2R2B knockdown and overexpression.** (A, B) PPP2R2B was silenced in J82 cells (A) and overexpressed in T24 cells (B) (n =3; one-way ANOVA with Dunnett's test and unpaired, 2-tailed t test, respectively). Three independent experiments were performed. Error bars were represented as mean ± SD.

**Fig. S2**


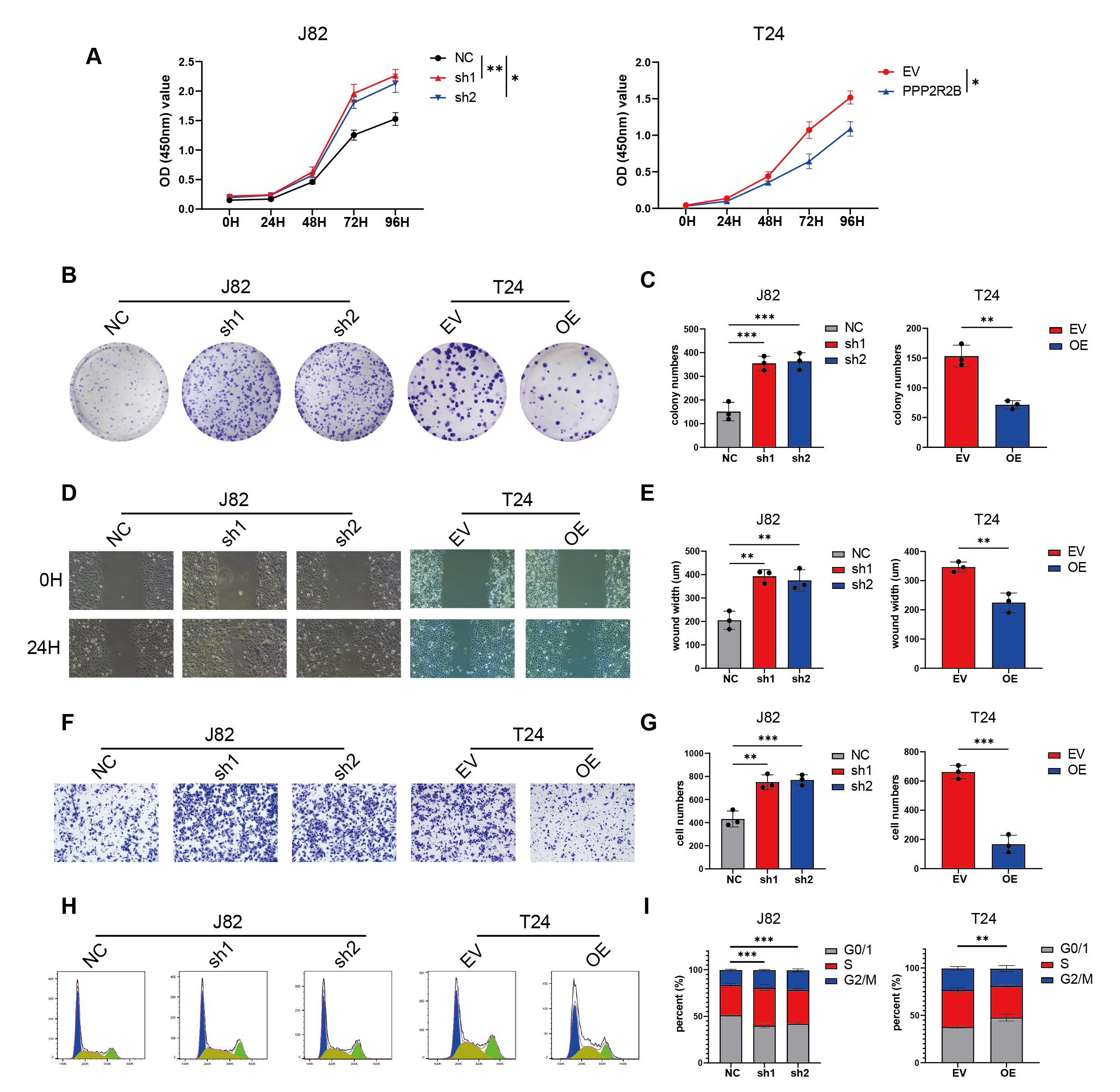


**Fig. S2 PPP2R2B inhibits the malignant phenotype of bladder cancer (BC) in vitro.** (A-C) PPP2R2B-knockdown J82 cells and PPP2R2B-overexpressing T24 cells were subjected to CCK-8 (A) (n = 3; two-way ANOVA with Dunnett's test and Bonferroni's test) and colony formation (B, C) assays (n = 3; one-way ANOVA with Dunnett's test and unpaired, 2-tailed t test). (D-G) Wound-healing (D, E) and transwell migration (F, G) assays using PPP2R2B-knockdown J82 cells (n = 3; one-way ANOVA with Dunnett's test) and PPP2R2B-overexpressing T24 cells (n = 3; unpaired, 2-tailed t test). (H, I) Cell cycle analysis indicating the percentages of PPP2R2B-silenced J82 cells (n = 3; two-way ANOVA with Tukey's test) and PPP2R2B-overexpressing T24 cells (n = 3; two-way ANOVA with Bonferroni's test) at different cell cycle phases. Three independent experiments were performed. Error bars were represented as mean ± SD.

Fig. S3


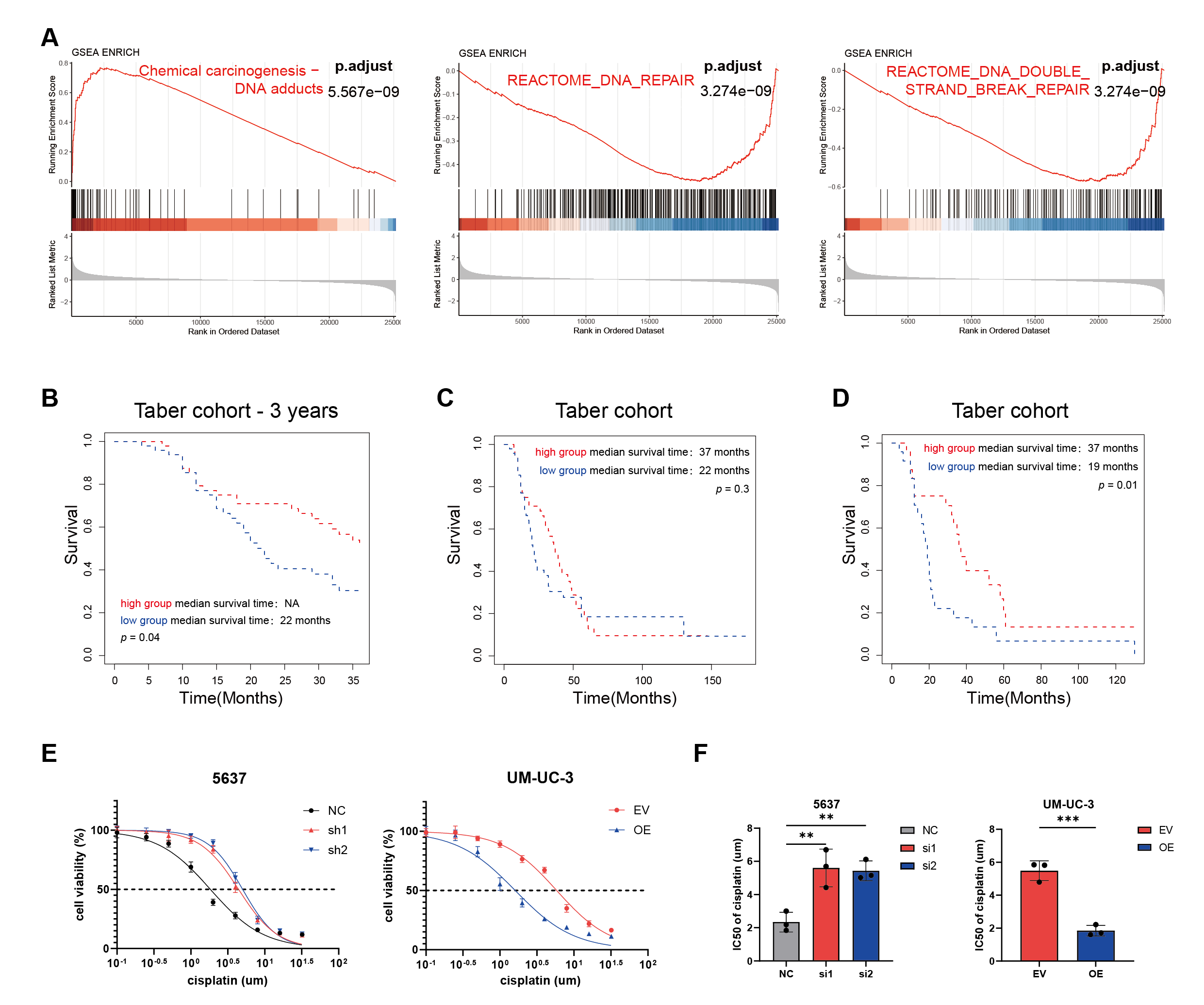


**Fig. S3 PPP2R2B is related with DNA damage repair.** (A) Gene set enrichment analysis in high and low PPP2R2B expression groups, according to median expression in tumors. (B, C) Survival analysis of patients with different PPP2R2B expression levels in Taber bladder cancer (BC) cohort, using median expression level as the cut-off value. (D) Survival analysis of patients with different PPP2R2B expression levels in Taber BC cohort, using quartile expression level as the cut-off value. (E, F) IC50 values of cisplatin in PPP2R2B-knockdown 5637 cells (n = 3; one-way ANOVA with Dunnett's test) and PPP2R2B-overexpression UM-UC-3 cells (n = 3; unpaired, 2-tailed t test).

Fig. S4


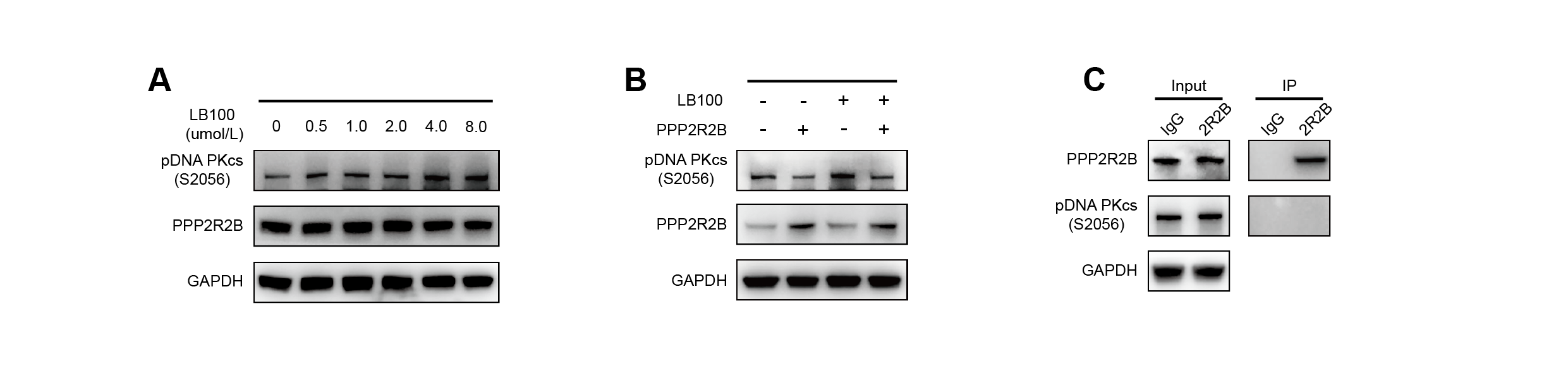


**Fig. S4 The regulation of DNA PKcs by PPP2R2B is largely independent of PP2A.** (A) Western blot analysis of pDNA PKcs (S2056) levels in T24 treated with PP2A-specific inhibitor LB100. (B) Detection of the pDNA PKcs (S2056) by western blot after PPP2R2B overexpression and LB100 treatment. (C) Western blot to detect the interaction between PPP2R2B and pDNA PKcs (S2056) after Co-IP.

Fig. S5


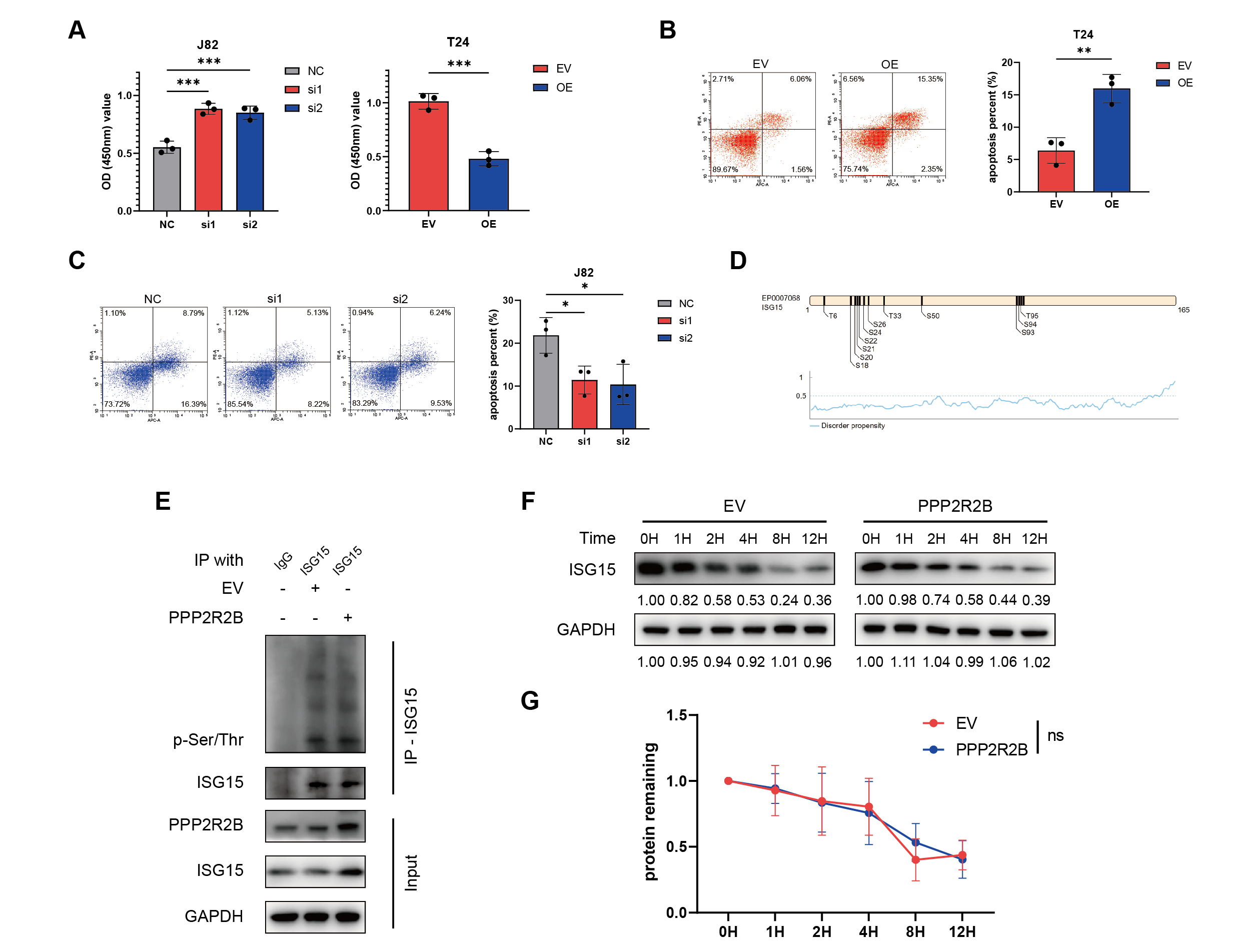


**Fig. S5 The role of ISG15 in BC chemoresistance and the effect of PPP2R2B on ISG15.** (A-C) Cell viability (A) and apoptosis (B, C) assays to evaluate the effect of ISG15 depletion (n = 3; one-way ANOVA with Dunnett's test) and overexpression (n = 3; unpaired, 2-tailed t test) in BC cells treated with cisplatin. (D) Illustration of the phosphorylation site of ISG15 in the Eukaryotic Phosphorylation Site Database. (E) Detection of the phosphorylation of ISG15 by western blot after PPP2R2B overexpression. (F, G) Rate of ISG15 degradation treated with Cycloheximide (CHX) when PPP2R2B was overexpressed or not. Three independent experiments were performed. Error bars were represented as mean ± SD.

Fig. S6

**
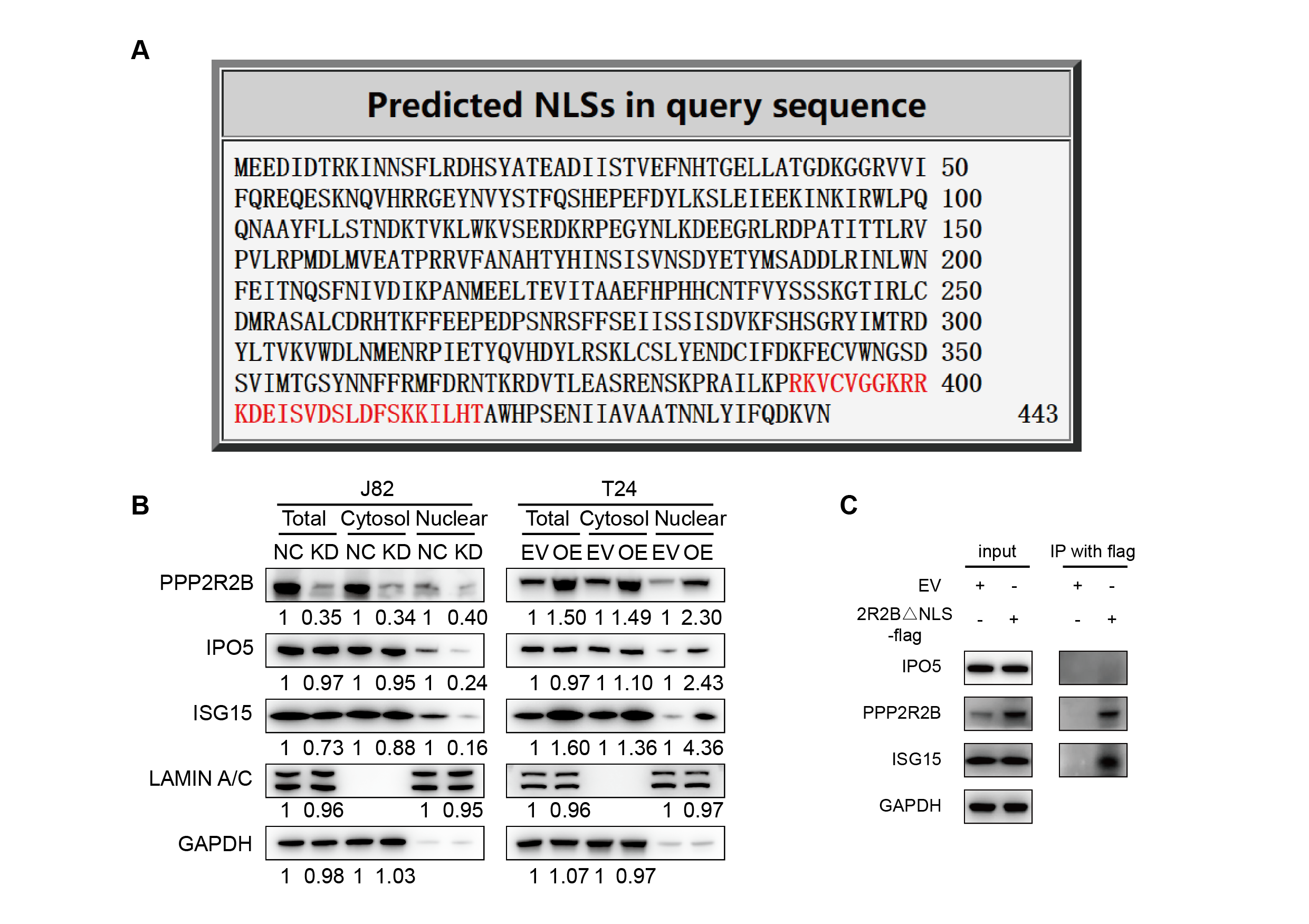
**

**Fig. S6 The NLS sequence of PPP2R2B is essential for interacting with IPO5.** (A) Nuclear localization signal (NLS) in PPP2R2B is predicted using cNLS Mapper. (B) Subcellular distribution of ISG15 detected by western blot after PPP2R2B knockdown and overexpression. (C) Western blot to detect the interaction between 2R2BΔNLS and IPO5/ISG15 after IP of flag. Three independent experiments were performed.

Fig. S7


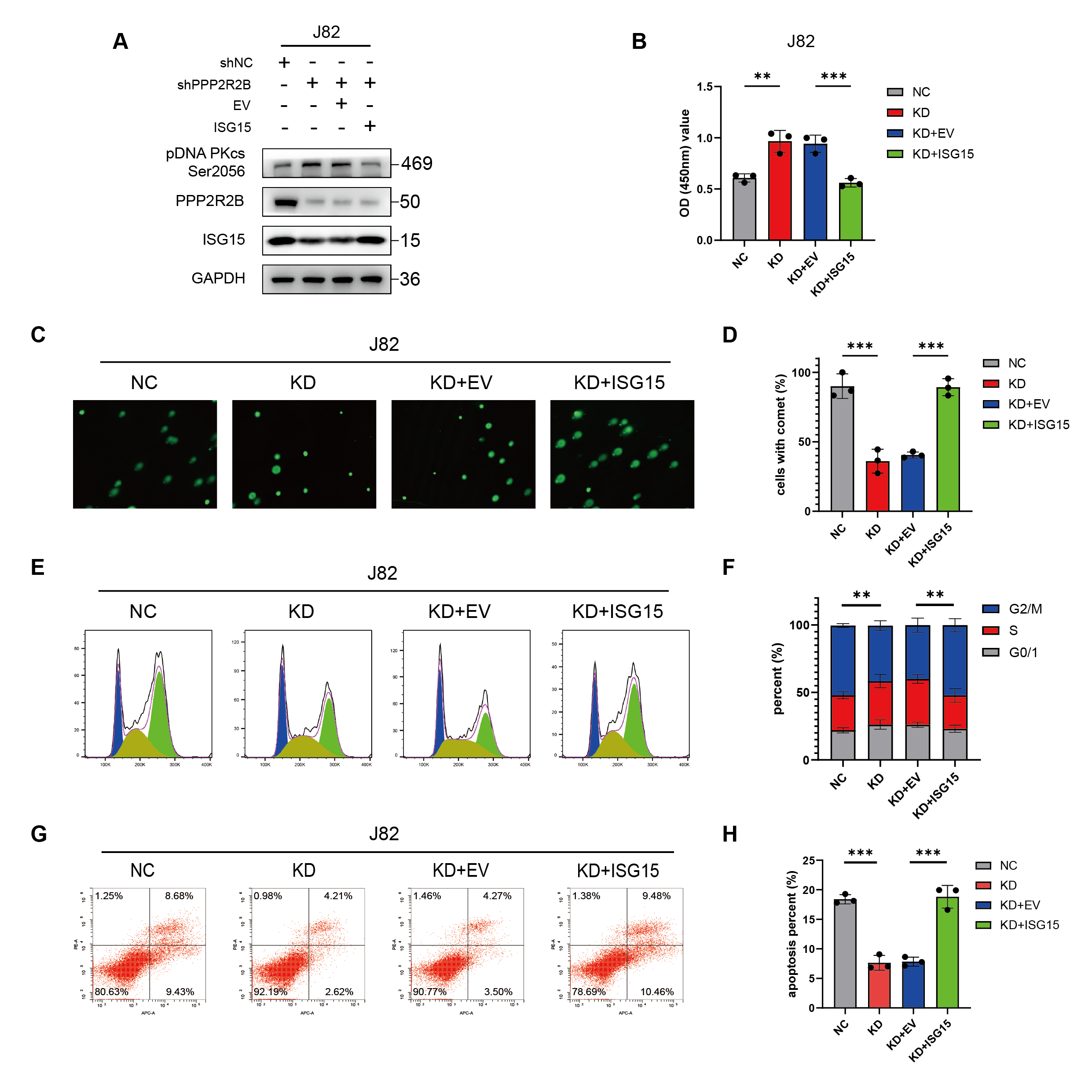


**Fig. S7 ISG15 is a core molecule in PPP2R2B-regulated sensitivity to cisplatin.** (A) Overexpression of ISG15 inhibited PPP2R2B silencing-mediated up-regulation of pDNA PKcs (Ser2056). (B–H) Cell viability (B) (n = 3, one-way ANOVA with Tukey's test), comet (C, D) (n = 3, one-way ANOVA with Tukey's test), cell cycle (E, F) (n = 3, two-way ANOVA with Tukey's test), and apoptosis (G, H) (n = 3, one-way ANOVA with Tukey's test) assays to evaluate the effect of ISG15 overexpression on PPP2R2B-knockdown in J82 cells treated with cisplatin. Three independent experiments were performed. Error bars were represented as mean ± SD.

Fig. S8


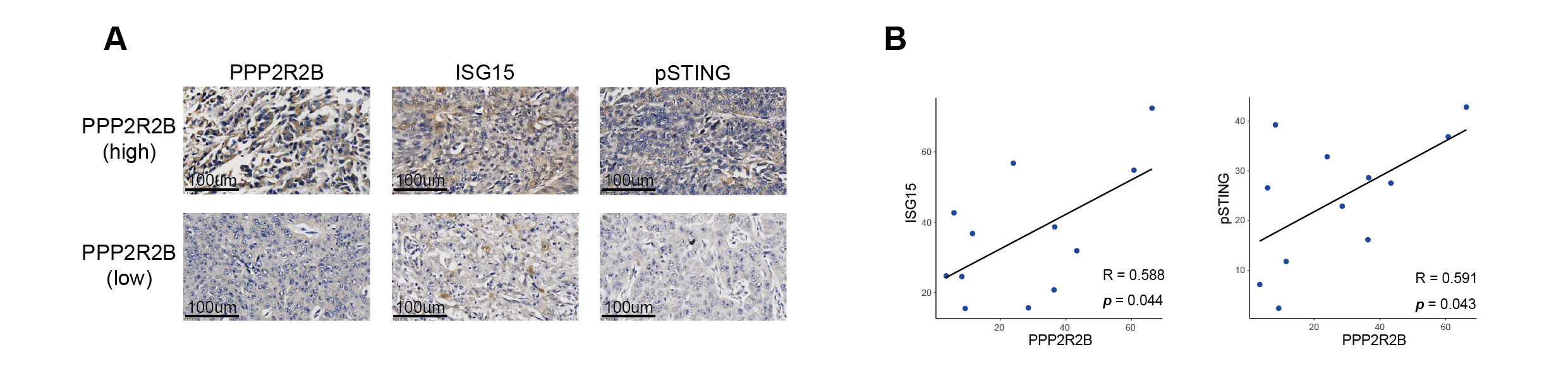


**Fig. S8 IHC staining of PPP2R2B/ISG15/STING axis in clinical samples.** (A) Detection of PPP2R2B/ISG15/pSTING expression levels by IHC in BC. (B) Pearson correlation between PPP2R2B/ISG15/pSTING IHC score.

Fig. S9


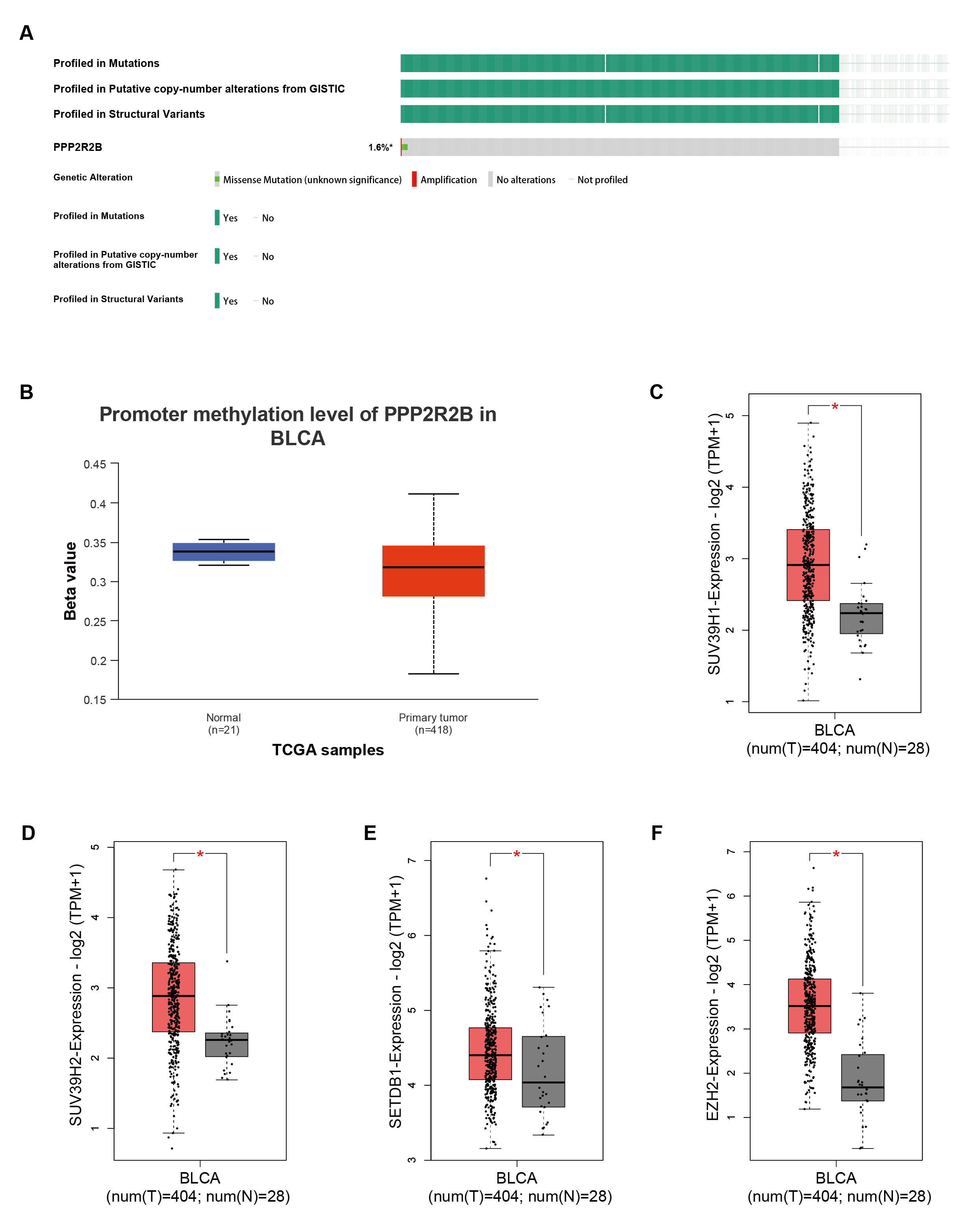


**Fig. S9 Exploration of PPP2R2B upstream regulation using bladder cancer (BC) data from public databases.** (A) Few PPP2R2B mutations are present in The Cancer Genome Atlas (TCGA) database. (B) PPP2R2B promoter DNA methylation levels were not high in BC. (C–F) Comparison of SUV39H1/2 (C, D), SETDB1 (E), and EZH2 (F) expression levels between BC and normal bladder tissue in TCGA BC cohort.

Fig. S10


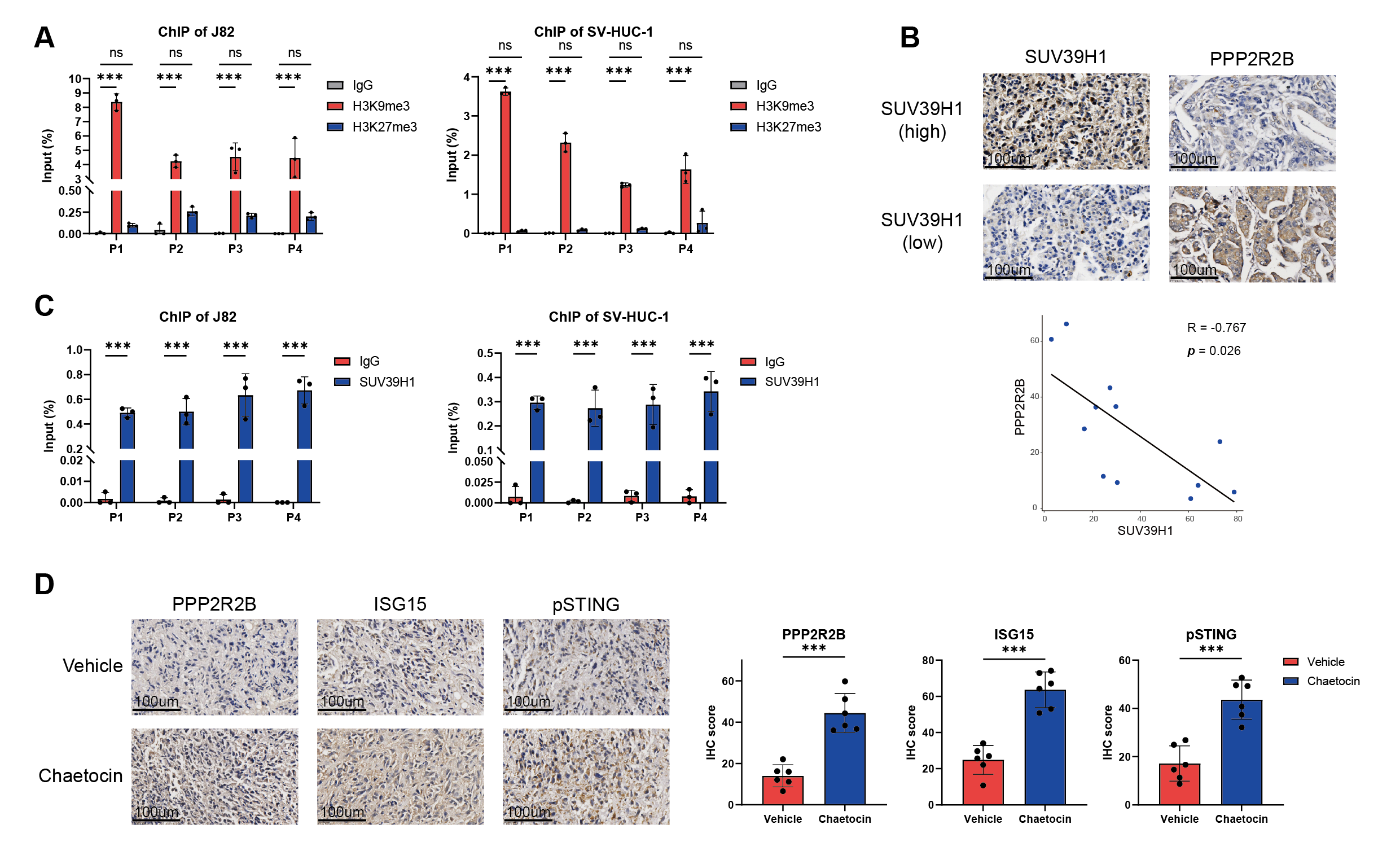


**Fig. S10 The verification of SUV39H1/PPP2R2B/ISG15/STING axis.** (A) ChIP-qPCR of H3K9me3- and H3K27me3-enriched samples at the PPP2R2B promoter in J82 and SV-HUC-1 cells (n = 3; two-way ANOVA with Dunnett's test). (B) Detection of SUV39H1/PPP2R2B expression levels by IHC in BC and the pearson correlation between SUV39H1/PPP2R2B IHC score. (C) ChIP-qPCR of SUV39H1-enriched samples at the PPP2R2B promoter in J82 and SV-HUC-1 cells (n = 3; two-way ANOVA with Bonferroni's test). (D) Detection of PPP2R2B/ISG15/pSTING expression levels by IHC in xenograft tumors and the comparison of the IHC score (n = 6; unpaired, 2-tailed t test).

| **Table S1 The sequences of siRNA, shRNA and primers.** | |
| --- | --- |
| **siRNA** | **sequences** |
| siPPP2R2B-1 | GGAAGATCCAAGCAACAGA |
| siPPP2R2B-2 | CCTGAAGAGTTTAGAAATA |
| siISG15-1 | CTGAGCATCCTGGTGAGGAAT |
| siISG15-2 | CATGTCGGTGTCAGAGCTGAA |
| siSUV39H1-1 | GCATCACTGTAGAGAATGA |
| siSUV39H1-2 | GGGTCCGTATTGAATGCAA |
| siSUV39H2 | GGAGATATATCTTCAGATT |
| siSETDB1 | CAAAGATGGTGACCTGATA |
| siSETDB2 | GCCCAAATCTCTTGGTACA |
|  |  |
| **shRNA** |  |
| shPPP2R2B-1 | GACATTATCTCTACGGTAGAA |
| shPPP2R2B-2 | GAAATTATCTCTTCGATTTCG |
|  |  |
| **Primers** |  |
| PPP2R2B-F | CCATGAACCCGAGTTCGATTAC |
| PPP2R2B-R | GGCCCTCCTCATCTTTCAGATT |
| ACTB-F | CATGTACGTTGCTATCCAGGC |
| ACTB-R | CTCCTTAATGTCACGCACGAT |
| ISG15-F | CGCAGATCACCCAGAAGATCG |
| ISG15-R | TTCGTCGCATTTGTCCACCA |
| IFNB1-F | ATGACCAACAAGTGTCTCCTCC |
| IFNB1-R | GGAATCCAAGCAAGTTGTAGCTC |
| SETDB1-F | GAAGTCCCGAGTTGAGGAGG |
| SETDB1-R | CTCCTCACAGCACCCATATTT |
| SETDB2-F | TCAAAGATGGGTCTGCCACC |
| SETDB2-R | CACAGGCATAGGATCCTTTATTGA |
| SUV39H1-F | CCTGCCCTCGGTATCTCTAAG |
| SUV39H1-R | ATATCCACGCCATTTCACCAG |
| SUV39H2-F | TCTATGACAACAAGGGAATCACG |
| SUV39H2-R | GAGACACATTGCCGTATCGAG |
|  |  |
| **PPP2R2B primers for ChIP** | |
| P1-F | TGTCCAAAACGAAGTGCAAA |
| P1-R | ACACAGGTGGAGGAAAGCAC |
| P2-F | GTAGACGTGGCCCTTAGCTG |
| P2-R | AGCGACTAGCTTGCAGGTTC |
| P3-F | AGAAAGGCACCATTTTGTCG |
| P3-R | GGAGATGCCCAACAGGTTC |
| P4-F | TACCCGCAAAATCAACAACA |
| P4-R | AAGCACAGTGATCCGCAACT |
